# Multireference Theory of Scanning Tunneling Spectroscopy Beyond One-Electron Molecular Orbitals: Can We Image Molecular Orbitals?

**DOI:** 10.1021/jacs.5c08166

**Published:** 2025-07-02

**Authors:** Manish Kumar, Diego Soler-Polo, Marco Lozano, Enzo Monino, Libor Veis, Pavel Jelinek

**Affiliations:** † Institute of Physics, Czech Academy of Sciences, Prague 16200, Czech Republic; ‡ Department of Condensed Matter Physics, Faculty of Mathematics and Physics, Charles University, Prague 2 CZ12116, Czech Republic; § Department of Theoretical Chemistry, J. Heyrovsky Institute of Physical Chemistry, Czech Academy of Sciences, Prague 18200, Czech Republic; ∥ Czech Advanced Technology and Research Institute (CATRIN), Palacký University Olomouc, Olomouc 779 00, Czech Republic

## Abstract

Recent progress in on-surface chemistry has enabled the synthesis of novel polyradical molecules with interesting electronic structure, which are hardly available in solution chemistry. Moreover, the possibility to characterize their electronic structure with scanning tunneling spectroscopy (STS) with the unprecedented spatial resolution opens new possibilities to understand their nontrivial electronic structure. However, experimental STS maps of molecules on surfaces are interpreted using one-electron STM theory within the framework of one-electron molecular orbitals nowadays. Although this standard practice often gives relatively good agreement with experimental data for closed-shell molecules, it fails to address multireference polyradical molecules. In this manuscript, we provide multireference STM theory including out-of-equilibrium processes of removing/adding an electron within the formalism of many-electron wave functions for the neutral and charged states. This can be accomplished by the concept of so-called Dyson orbitals. We will discuss the examples where the concept of Dyson orbitals is mandatory to reproduce experimental STS maps of polyradical molecules. Finally, we critically review the possibility of the experimental verification of the so-called SOMO/HOMO inversion effect using STS maps in polyradical molecules. Namely, we will demonstrate that experimental STS measurements cannot provide any information in case of strongly correlated molecules about the ordering of one-electron molecular orbitals and, therefore neither about the SOMO/HOMO inversion effect.

## Graphical abstract

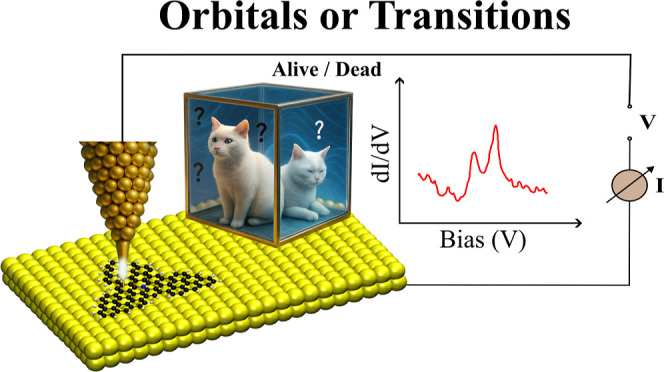

## Introduction

Scanning tunneling microscopy (STM)^1^ is a unique tool that allows us not only to image objects with atomic resolution, but also to obtain valuable information about the electronic structure.^2^ Namely, scanning tunneling spectroscopy (STS)^3,4^ provides information about the electronic structure of individual molecules^5^ on the surfaces of solids. The spatial d*I*/d*V* maps obtained by STS measurements are often interpreted using one-electron Frontier molecular orbitals.^6^ Moreover, in many cases, d*I*/d*V* maps are associated directly with images of molecular orbitals, due to the coincidence of their spatial distribution. However, this interpretation is in sharp contradiction with one of the basic postulates of quantum mechanics, which excludes direct observation of the wave function of a quantum system. Therefore, this approach has received a lot of criticism, especially from the chemistry community, see e.g. refs 7–9 . However, this dichotomy is very often defended in the STM community, due to the relatively good agreement between experimental data and calculated one-electron molecular orbitals, as a *philosophical* question. However, the interpretation of STS data based on one-electron molecular orbitals can be quite problematic, especially in the case of strongly correlated molecules. For example, the recent development of on-surface synthesis^10–18^ has enabled the preparation of highly radical molecules with strongly correlated electronic structures. As practice shows, it is precisely in these cases that the interpretation based on one-electron molecular orbitals often fails.^16,19,20^ Moreover, recently, molecules exhibiting an unusual arrangements of molecular orbitals, where the energy level of the HOMO orbital is higher than that of the SOMO orbital, have received considerable attention.^21–24^ This effect, called SOMO/HOMO inversion (SHI), has recently been discussed in connection with STM measurements of diradical molecules.^25^

We should note that the problem of the correct interpretation of d*I*/d*V* maps discussed here was already addressed by several groups before.^19,20,26^ But we feel that a concise picture that is accessible also for general audience, including a simple guideline for the interpretation, is still missing. Here we introduce the concept of multireference Dyson orbitals,^27,28^ which enable us to describe the nonequilibrium process during the single electron tunneling through the molecules simultaneously considering the strongly correlated electronic structure of the polyradical molecules.

The article is organized as follows. First, we briefly recapitulate the concept of one-electron molecular orbitals. We summarize the one-electron theory describing tunneling in STM junction under quasi-equilibrium conditions and show the connection with one-electron molecular orbitals. Next, we introduce the concept of Dyson orbitals, the importance of which we demonstrate on selected cases of strongly correlated molecules. Namely, we make a comparison with available experimental STS measurements. Finally, we critically discuss the possibility of detecting the SHI effect using STS measurements.

## Results and Discussion

### The Concept of Molecular Orbitals

For the sake of simplicity, we briefly summarize the concept of one-electron molecular orbitals (MO), which plays a fundamental role in our understanding of the physical and chemical properties of molecular compounds. They represent a spatial localization of individual electrons in molecule, but they are not uniquely defined quantities. Strictly speaking, MOs are mathematical objects that represent eigenfunctions of a given one-electron quantum operator. A complete description of an electron must include its spin, therefore, the fundamental one-electron functions correspond to molecular spin–orbitals. When referring only to their spatial component, they are referred to as MOs. In the case of canonical Kohn–Sham (KS) spin orbitals, $\phi_{i}^{KS}\left(x\right)$ , they are obtained in the framework of density functional theory (DFT) solving the set of one-electron Kohn–Sham equations of the *N*-electron system^29^

1 $$\left(-\frac{\hbar }{2m}\nabla^{2}+V_{KS}\left(x\right)\right)\phi_{i}^{KS}\left(x\right)=\epsilon_{i}\phi_{i}^{KS}\left(x\right)$$

where, *V*_KS_ represents an effective potential acting on *i*-th noninteracting electron, where *i* = 1, ..., *N*. The variable **x** = (**r**,σ) labels both position **r** and spin σ. Variable ϵ_*i*_ represents the spin orbital energy of the corresponding one-electron KS canonical spin orbital $\phi_{i}^{KS}$ . In the DFT framework, the many-electron ground state Ψ^KS^(**x**_1_,··· ,**x**_*N*_) of the *N*-electron system is described as a single determinant of the *N* lowest KS spin orbitals $\phi_{i}^{KS}\left(x\right)$

2 ΨKS(x1,...,xN)=1N!×|ϕ1KS(x1)ϕ2KS(x1)···ϕNKS(x1)ϕ1KS(x2)ϕ2KS(x2)···ϕNKS(x2)⋮⋮⋱⋮ϕ1KS(xN)ϕ2KS(xN)···ϕNKS(xN)|.

Note that the KS many-electron ground state Ψ^KS^ is a single-reference state, as it is described by a single Slater determinant (eq 2). Therefore, it fails to describe strongly correlated open-shell systems as will be discussed later on. To describe correctly strongly correlated open-shell systems of *N*-electrons, we have to used multireference many-electron wave function Ψ­(**x**_1_,··· ,**x**_*N*_), which consist of linear combinations of several Slater determinants with non-negligible contributions to the ground state.

For clarity, throughout this article, we employ small Greek letters (ϕ, φ, χ···) to denote single-particle wave functions (i.e., orbitals) and capital Greek letters (i.e., Ψ) to denote many-electron states.

Natural orbitals ϕ_*i*_^NO^(**r**) are another type of MOs, which are known to provide the most compact representation of correlated wave functions.^30^ The set of *N* natural orbitals are eigenvectors obtained from diagonalization of the spinless 1-particle reduced density matrix γ­(**r**, **r**′)

3 $$\int \gamma \left(r,r^{\prime }\right)\phi_{j}^{NO}\left(r^{\prime }\right)dr^{\prime }=\lambda_{j}\phi_{j}^{NO}\left(r\right)$$

where the density matrix γ­(**r**, **r**′) is defined in the position representation

4 γ(r,r′)=N∑σ,σ′∫Ψ*(rσ,x1,...,xN−1)×Ψ(r′σ′,x1,...,xN−1)×dx1···dxN−1.

The positive eigenvalues λ_*j*_ in eq 3 represent the occupations of the natural orbitals ϕ_*j*_^NO^. The occupied and unoccupied natural orbitals have the occupancy λ_*j*_ close to 2 or 0, respectively. The natural orbitals ϕ_*j*_^NO^ whose occupations have fractional values significantly different from integer values of 2 or 0 contribute to the number of unpaired electrons, where λ = 1 means one whole unpaired electron (i.e., greatest radical character for the given orbital).

Unitary transformations can convert delocalized canonical orbitals into localized molecular orbitals (LMOs) and vice versa. LMOs help bridge molecular Lewis structures with quantum-chemical calculations, facilitate the interpretation of correlations between molecular fragments,^14^ and enable a more efficient treatment of dynamic electron correlation.^31^

It should be stressed that the one-electron MOs are experimentally not observable. According to the basic postulates of Quantum Mechanics, the *observables* of a system are self-adjoint operators *A*, acting on physical states Ψ. The *measurable* quantities are the eigenvalues of such operators represented in a given basis set. If a many-electron wave function Ψ describing a given system has single-reference character, i.e. can be approximated by a single Slater determinant of a set of MOs, {ϕ}, the expected value of observable *A*, denoted by ⟨*A*⟩, can be expressed as

5 $$\left\langle A\right\rangle =\sum_{j}\left\langle \phi_{j}\left|A\right|\phi_{j}\right\rangle$$

This explains why one-electron MOs ϕ can be used as tools to compute experimentally observable quantities. However, as such, the one-electron MOs ϕ themselves are purely mathematical objects that do not have any physical meaning. Hence, MOs are not directly observable, which contradicts the general practice where d*I*/d*V* measurements obtained from STM are interpreted on the basis of one-electron MOs.

### Bardeen Perturbation Theory of Tunneling

The generally adopted theory of scanning probe microscopy to describe the tunneling process between tip and sample is based on the time-dependent first-order perturbation theory.^2^ For detailed derivation of the tunneling theory, we refer readers to the excellent book by C. J. Chen.^2^ The theory assumes the resonant tunneling process between tip and sample both represented by the ground state wave functions. Moreover, it also considers the quasi-equilibrium situation, where the applied bias voltage eV between tip and sample causes only a rigid shift of the density of states of the sample ρ_*s*_(*E*_F_ – eV) with respect to the density of states of the tip ρ_*t*_(*E*_F_). The conductance $G=\frac{dI}{dV}$ at a particular applied bias voltage eV between tip and sample can be expressed as follows

6 $$\frac{dI}{dV}=\frac{4\pi e}{\hbar }\rho_{s}\left(E_{F}-eV\right)\rho_{t}\left(E_{F}\right){\left|M_{ts}\right|}^{2}$$

*E*_F_ means the Fermi energy. The transition matrix between tip and sample *M*_*ts*_ is expressed by Bardeen’s surface integral

7 $$M_{ts}=-\frac{\hbar^{2}}{2m}\int_{S}\left(\phi_{t}^{*}\nabla \phi_{s}-\phi_{s}\nabla \phi_{t}^{*}\right)dS$$

where ϕ_*t*,*s*_ represents one-electron wave functions of tip and sample, respectively, and *m* is the mass of the electron. The matrix element *M*_*ts*_ is defined by the selection rules imposed by the symmetry of the tip ϕ_*t*_ and sample ϕ_*s*_ wave functions.

In the so-called Tersoff–Hamann approach, eq 6 can be further simplified, provided that the tip wave function ϕ_*t*_ can be represented by a single s-like orbital and the constant density of state of tip ρ_*t*_(*E*) ≈ *const*.^33^ Thus, the tunneling current is proportional to the local density of the states of the surface ρ_*s*_, and eq 6 can be simplified as follows

8 $$\frac{dI}{dV}\approx \rho_{s}\left(\epsilon -eV\right)$$

In the case of physisorbed molecules on metal surfaces with negligible hybridization and charge transfer between the molecule and surface, the density of states ρ_*s*_ can be approximated by the set of canonical or natural one-electron molecular orbitals typically obtained by solving one-electron Kohn–Sham equations.^29^ Thus, for particular bias voltages, the density of states ρ_*s*_ reduces to a particular canonical orbital, which is in resonance with the Fermi level of the tip; i.e., ρ_*s*_ = |ϕ_*i*_|^2^. Consequently, d*I*/d*V* measurements within the Tersoff–Hamann approach can be directly associated with a particular molecular orbital ϕ_*i*_. This is a remarkable conclusion, which is often used by SPM community to justify the interpretation of the experimental d*I*/d*V* maps within the framework of one-electron molecular orbitals. However, in cases when the molecule is chemisorbed or strongly hybridized with the substrate, the substrate can significantly alter its electronic structure. Capturing these effects remains challenging due to high computational cost, especially in the framework of computational methods beyond single-reference DFT calculations.

Indeed, the long-standing experience shows that such association with molecular orbitals can often provide a reasonable agreement with the experimental evidence. For example, in the seminal work of Repp et al.,^6^ they showed that spatial STM maps acquired at given low bias voltages on pentacene molecules with a metallic tip (see Figure 1a) exhibit striking agreement with the one-electron canonical highest occupied molecular orbital (HOMO) and lowest unoccupied molecular orbital (LUMO) obtained from DFT (see Figure 1c) as well as their corresponding d*I*/d*V* maps simulated with tip represented by s-like orbital (see Figure 1b).

**Figure 1:**
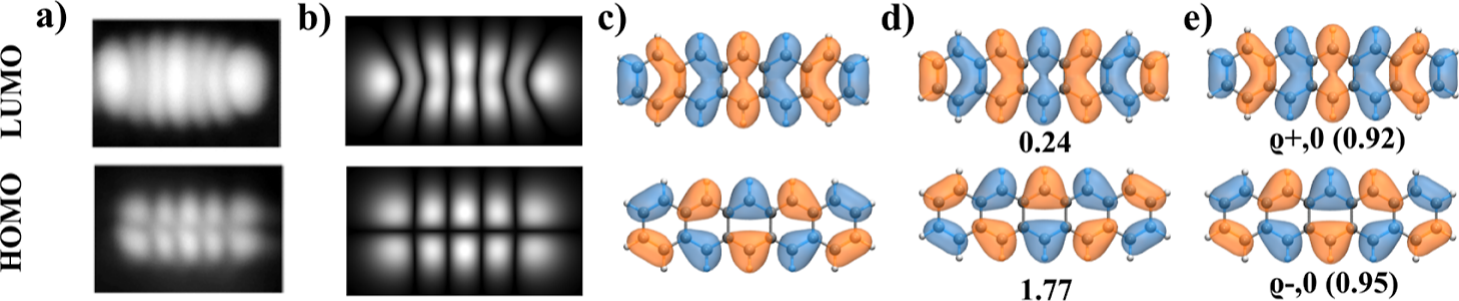
(a) Experimental STM maps adopted with permission from ref 6 Copyright 2005 American Physical Society; (b) simulated d*I*/d*V* maps of canonical DFT orbitals obtained from spin-unpolarized DFT-PBE0 calculations using PP-STM code with metal-like tip;^32^ (c) canonical DFT orbitals obtained from spin-unpolarized DFT-PBE0 calculations; (d) multireference natural orbitals obtained from CASCI­(12,12) calculations with corresponding occupation; (e) multireference Dyson orbitals obtained from CASCI­(12,12) calculations with corresponding strengths for the pentacene molecule.

According to the multireference complete active space configuration interaction (CASCI) calculations employing the active space of 12 electrons in 12 orbitals, CASCI­(12,12), the ground state wave function Ψ_*o*_ of the neutral pentacene molecule has a dominant contribution of a single Slater determinant with a doubly occupied HOMO orbital, see middle Figure 2a. Consequently, the occupancy of the natural Frontier HONO and LUNO orbitals is 1.77 and 0.24, respectively (see in Figure 1d), indicating the closed-shell character of the pentacene molecule. Comparison of Figure 1c,d reveals a very similar character of one-electron canonical DFT orbitals and natural orbitals obtained from multireference CASCI­(12,12) calculations.

**Figure 2:**
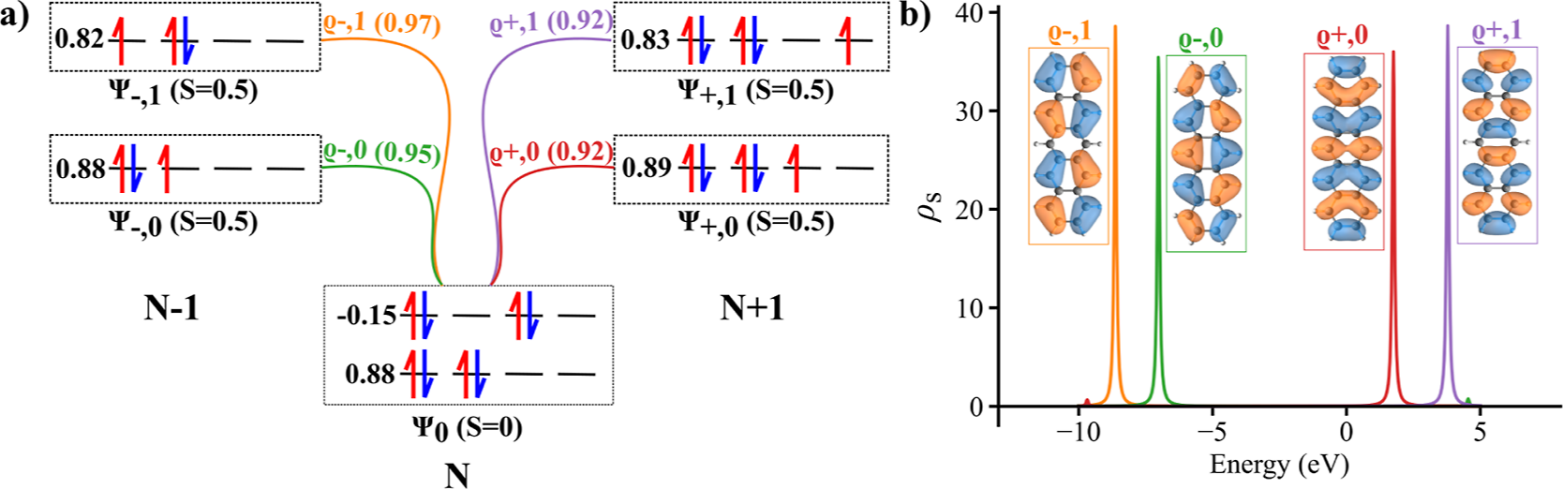
(a) Multireference wave functions of neutral ground state and charge state multiplet. Horizontal lines represent individual molecular orbitals (MOs) solely for occupancy purposes and do not indicate degeneracy. This format is consistently used throughout the article. (b) Multireference spectral function for the pentacene molecule.

This example demonstrates that the d*I*/d*V* maps of weakly bound molecules on surfaces with a strong closed-shell character can often be reasonably well interpreted on the basis of one-electron MOs. This experience led to a common practice that associates the spatial distribution of d*I*/d*V* maps of weakly bound molecules on surfaces with Frontier one-electron MOs using the Tersoff–Hamann approach. In this context, the criticism of this approach could be viewed as a simple philosophical discussion that does not have any implications for the correctness of the interpretations using the association with the concept of one-electron MOs. However, as frequently happens, evil resides in some details, as will be discussed later on.

### Concept of Dyson Orbitals

The STM perturbation theory sketched in the previous section is limited to a one-electron formalism at quasi-equilibrium conditions. Therefore, the perturbation formalism has two drawbacks. First, it cannot fully capture the tunneling process through strongly correlated molecular systems, where the one-electron picture fails. In the case of open-shell strongly correlated molecules, we employed multireference CASCI methods to correctly describe their electronic structure. Second, it does not explicitly consider a temporal addition/removal of an electron in the molecule during the tunneling process between tip and sample. In other words, the change of electron density associated with the transition from the ground state of the neutral molecule Ψ^*N*^ to the charged states Ψ^*N*±1^ caused by the tunneling of electrons through the molecule during the measurement is completely neglected.

To describe the transition from the neutral ground state to low-energy charged (*N* ± 1) states of strongly correlated molecules, we use the concept of so-called Dyson orbitals.^28^ We consider multireference wave functions of the ground state of the neutral molecule Ψ_0_(**x**_1_, ..., **x**_*N*_), and a manifold of low-energy states for the negatively and positively charged system Ψ_+,*j*_(**x**_1_, ..., **x**_*N*_, **x**_*N*+1_), Ψ_–,*j*_(**x**_1_, ..., **x**_*N*–1_). Dyson spin orbitals are then defined as the overlaps between the neutral ground state Ψ_0_ and *j*-th charged states Ψ_±,*j*_

9 ϱ+,j(x)=⟨Ψ+,j|ψ̂†(x)|Ψ0⟩=N+1∫Ψ0(x1,...,xN)*×Ψ+,j(x,x1,...,xN)dx1···dxN,ϱ−,j(x)=⟨Ψ−,j|ψ̂(x)|Ψ0⟩=N∫Ψ0(x,x1,...,xN−1)*×Ψ−,j(x1,...,xN−1)dx1···dxN−1.

Here, ${\mathrm{\psi ̂}}^{†}\left(x\right)$ and ψ̂(x) are the field operators for the creation and annihilation of an electron at position (**x**). Importantly, Dyson orbitals represent the transition amplitudes from the neutral ground state to a particular charged state *N* ± 1. Therefore, we can construct the multireference spectral function ρ_*s*_, which represents the local density of states of the molecular system upon charging by a single electron as follows

10 ρs(x,ω)=η∑j|⟨Ψ+,j|ψ̂†(x)|Ψ0⟩|2(ω−Ej)2+η2+η∑j|⟨Ψ−,j|ψ̂(x)|Ψ0⟩|2(ω−Ej)2+η2,

where *E*_*j*_ is the energy difference of the charged states |Ψ_±,*j*_⟩ with respect to the neutral ground state, |Ψ_0_⟩, ω is the energy and η is the broadening of the resonance peaks, linked to a finite lifetime of the excitations. Thus, the density of state ρ_*s*_(**x**, ω) is directly related to the Dyson orbitals. By substituting eq 10 defines the multireference density of states in the Tersoff-Hamann formula given by eq 8. We see that the spatial distribution of the d*I*/d*V* maps is directly determined by the Dyson orbitals. From this point of view, Dyson orbitals are superior to single-electron MOs, because they include the transition probabilities between neutral and charged multireference states, properly describing strongly correlated molecular systems. So the Dyson orbitals are observable because they remain invariant of the chosen basis set in which they are constructed. This can be demonstrated in the case of the benzene molecule as discussed in detail in the Supporting Information.

In the case of single-reference closed-shell molecules, it can be shown that Dyson orbitals reduce to single-electron canonical Frontier orbitals.^28^ If we employ the one-electron molecular orbital picture, the wave functions are described by a single Slater determinant and these integrals reduce to one-particle integrals. If the basis of orbitals is the same for neutral and charged systems, then ϱ_+,*j*_, ϱ_–,*j*_ coincide exactly with such one-electron canonical orbitals (see the Supporting Information for a detailed proof).

For clarity, let us discuss the case of the pentacene molecule to demonstrate the capability of the Dyson orbitals to describe the spatial d*I*/d*V* maps of closed-shell molecules. According to multireference CASCI­(12,12) calculation performed using the DFT orbitals shown in Figure S2, the ground state wave function Ψ_0_ of pentacene is predominantly represented by a single Slater determinant with a doubly occupied HOMO orbital, as shown in Figure 2a. The occupancies of the highest occupied natural orbital (HONO) and the lowest unoccupied natural orbital (LUNO) shown in Figure 1d confirm the closed-shell character of the pentacene molecule.

According to CASCI calculations, the ground Ψ_0,±_ and first excited Ψ_1,±_ states of charged (*N* ± 1) systems, displayed in Figure 2a, are well-described by a single-reference state as well. Consequently, the electron (de)­attachment during the tunneling process takes place exclusively in one MO, as shown schematically in Figure 2a.

Figure 2b shows the multireference spectral function ρ_*s*_(ω) of the pentacene molecule, including the corresponding lowest energy Dyson orbitals modeling the first ϱ_±,0_ and second ϱ_±,1_ positive/negative ionic resonances. Comparing Figure 1c–e, we see that the Dyson orbitals ϱ_±,0_ that model the first ionic resonances are almost identical with one-electron HOMO/LUMO orbitals. This observation can be rationalized by the aforementioned fact that the ground Ψ_±,0_ and first excited Ψ_±,1_ states of charged states corresponding to the (de)­attachment of an electron from the ground state Ψ_0_ of the neutral molecule can be well described by a single Slater determinant, see Figure 2a. Namely, the ground state Ψ_+,0_ of charge state *N* + 1 is represented by the Slater determinant with an extra electron in LUMO, while the ground state Ψ_–,0_ of *N* – 1 state by the Slater determinant with a missing electron in HOMO. Consequently, the single electron charging processes take place dominantly through HOMO/LUMO orbitals. This fact explains why single electron HOMO/LUMO orbitals may reproduce well d*I*/d*V* maps of pentacene molecule at the first ionization processes in both bias polarities.

For monoradical systems, DFT method typically describes the ground state accurately due to its single-reference character. However, the reliability of DFT for simulating ionic resonances depends on the nature of the electronic structure of the charged *N* ± 1 states. If they do not show the multireference character, MOs obtained from DFT calculations may approximate d*I*/d*V* maps well.

### Polyradical Open-Shell Molecules and Dyson Orbitals

Next, we will discuss two examples of strongly correlated polyradical molecules where the concept of Dyson orbitals obtained from multireference wave functions is mandatory to explain available experimental d*I*/d*V* measurements. We do not explicitly include the effect of the substrate, as the studied molecules are physisorbed on Au(111) and show negligible hybridization. In this regime, gas-phase treatment is a reasonable approximation.

We begin with polyradical open-shell triradical aza-triangulene (**TTAT**) molecule, synthesized by Vegliante et al.^34^ on an Au(111) surface, which possesses a high-spin quartet (S = 3/2) ground state. Figure 3a,b illustrate calculated energy spectrum and corresponding canonical MOs obtained from spin-polarized DFT calculation using PBE0 exchange–correlation functional.^35^ Here, blue/red color denotes states in down/up spin channels, respectively. The expectation value of the total spin operator (⟨*S* ^2^⟩), is 4.05, showing moderate spin-contamination. According to this one-electron MO diagram, the first and second electron detachment, corresponding to positive ionic resonances (PIR), take place at the states labeled A1, which are energetically distinct, corresponding to the opposite spin channel. The subsequent electron detachments occur from A2MO followed by doubly degenerate E MOs. On the other hand, the first and second electron attachment, related to negative ionic resonances (NIR), are represented by A2 and E MOs, respectively.

**Figure 3:**
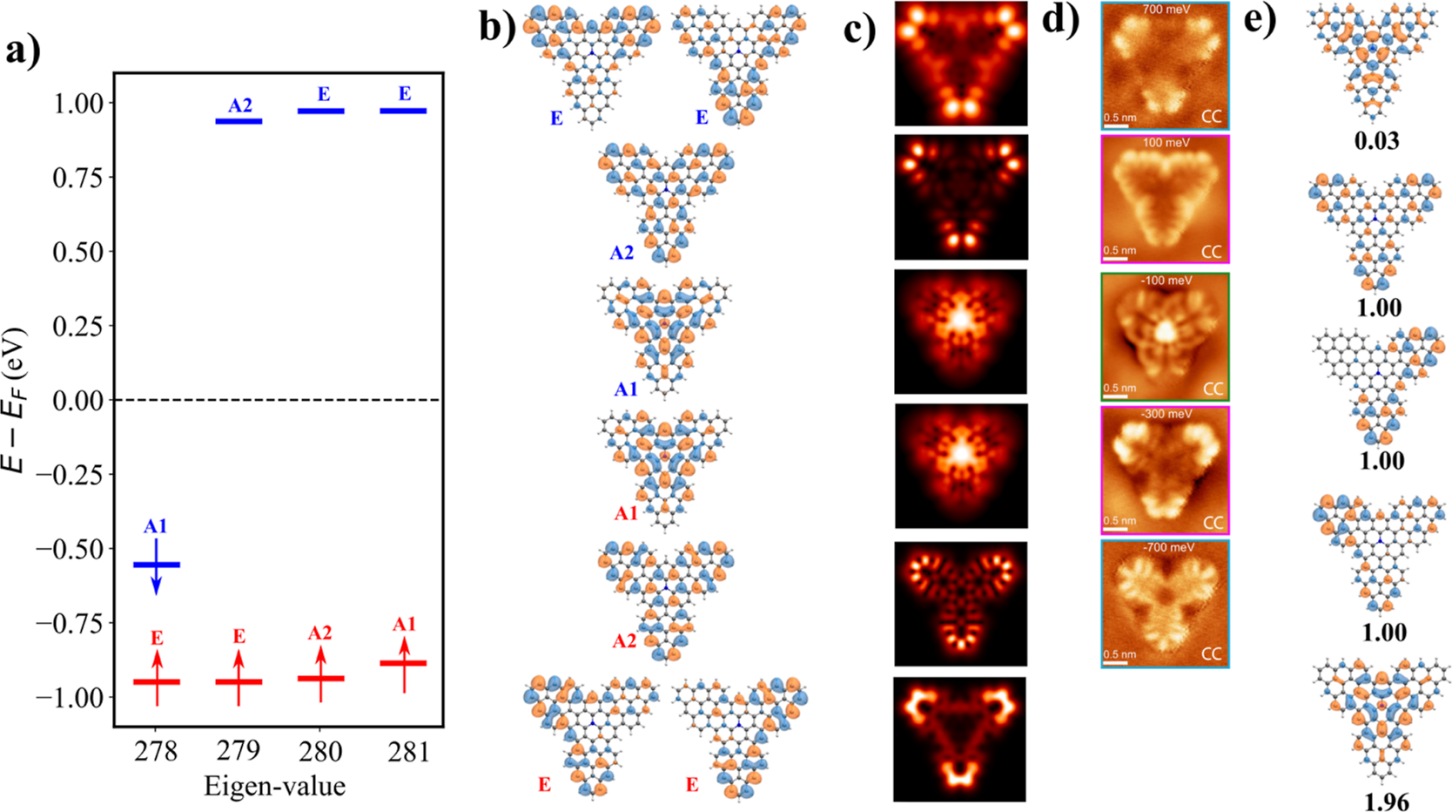
(a) Spin polarized DFT-PBE0 eigenvalue; (b) canonical DFT orbitals obtained from spin polarized DFT-PBE0 calculations; (c) simulated d*I*/d*V* maps of corresponding DFT-PBE0 orbitals; (d) experimental d*I*/d*V* maps adapted with permission from ref 34 Copyright 2025 American Chemical Society; (e) multireference natural orbitals obtained from CASCI­(11,11) calculation with corresponding occupation for **TTAT** molecule.

Figure 3c displays simulated d*I*/d*V* maps corresponding to canonical MOs shown in Figure 3b. If we compare them with the experimental d*I*/d*V* maps acquired for different ionic resonances (IR) shown in Figure 3d, we find a poor agreement between them. Only A1 SOMO is able to match with the experimental d*I*/d*V* map of the first PIR acquired at −100 meV. However, the second A1 orbital is completely missing. Also, in the case of doubly degenerate E and A2 SOMO states, their appearance in d*I*/d*V* maps seems to be exchanged. Similarly, the experimental d*I*/d*V* map of the first NIR acquired at 100 meV does not fit with the calculated d*I*/d*V* maps corresponding to A2 LUMO at all. Instead, the first NIR matches better two doubly degenerate E unoccupied orbitals. These discrepancies show that the one-electron canonical MOs cannot explain sufficiently the d*I*/d*V* maps of **TTAT** molecule.

We carried out CASCI calculations using the orbitals from restricted open-shell Kohn–Sham shown in Figure S3 to obtain multireference electronic states for neutral and charged **TTAT** molecule, which reveal the quartet ground state (*S* = 3/2) of neutral **TTAT** with three unpaired electrons according to the occupancies of NOs, shown in Figure 3e. Due to the presence of three unpaired electrons in the neutral state and four unpaired electrons in the negatively charged state, the wave function exhibits strong multireference character, as illustrated in Figure 4a. The ground-state wave function for the neutral molecule in the *S*_*z*_ = 1.5 subspace can be approximated by a single Slater determinant, as shown in Figure S6. However, for the negatively charged state, the presence of four unpaired electrons requires a multireference wave function for an accurate description.

**Figure 4:**
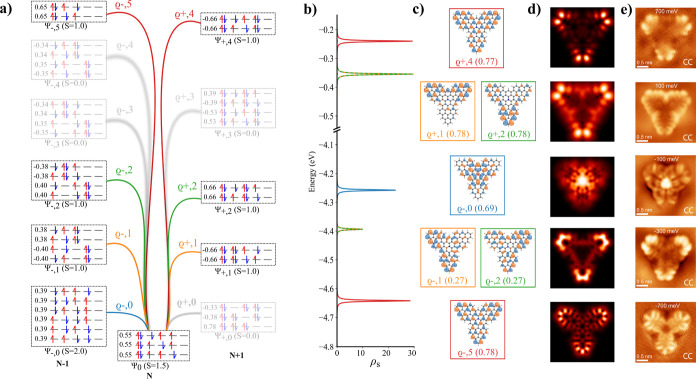
(a) Multireference wave functions of neutral ground state and charge state multiplets; (b) calculated multireference spectral function; (c) multireference Dyson orbitals obtained from CASCI­(11,11) calculations with corresponding strengths; (d) simulated d*I*/d*V* of corresponding Dyson orbitals using PP-STM code^32^ with CO-like tip; (e) experimental d*I*/d*V* maps adapted with permission from ref 34 Copyright 2025 American Chemical Society for **TTAT** molecule.

There is a question whether we can use multireference natural orbitals instead of Dyson orbitals to rationalize the experimental STM/STS maps. First, we should note that multireference NOs do not take into account transition probabilities from the neutral ground state Ψ_0_ to charged states Ψ_±,*j*_, as Dyson orbitals do. In the following, we will see that these transitions may have serious implications on the STS spectra. Second, natural orbitals do not possess energy ordering, as they are categorized by occupation numbers instead of energy levels. This inherent trait presents difficulties for employing natural orbitals in direct comparisons with experimental data, particularly in systems where precise energy-level alignment is imperative. As depicted in Figure 3e, there are three natural orbitals with an occupation of 1 that should appear in the first two ionization sequences. However, Figure 3d reveals that the initial ionization arises from one of the doubly natural orbitals. On the other hand, the ionization event at −100 meV is attributable to a blend of many SONO orbitals. From the discussion above, we see that multireference natural orbitals cannot be regarded as a substitute for Dyson orbitals when comparing d*I*/d*V* spectra with experimental maps.

Next, we calculated Dyson orbitals ϱ_±,j_ using the multireference state to describe the transition from the neutral ground state Ψ_0_ to charged states Ψ_±,*j*_ driven by the tunneling process, as shown in Figure 4a–c. The multireference electronic states are schematically represented by the expansion of the most important Slater determinants in the basis of close-shell DFT MOs. It is evident that the electronic structure of **TTAT** molecule is characterized by highly complex multireference states in both neutral and charged states, rendering the electronic properties and d*I*/d*V* maps challenging to elucidate via single-reference (DFT) methods.

Importantly, we observe that not all the transitions from the neutral quartet (*S* = 3/2) ground state Ψ_0_ are allowed. Namely, transitions to the singlet (*S* = 0) states are prohibited, due to the fact that the single electron tunneling cannot change the total spin of the final state more than *S* = ±1/2. The presence of the forbidden transition states strongly affects the resulting spectral function ρ_*s*_(ω) and the emergence of ionic resonances in experimental STS spectra.

Figure 4b,c, presents the multireference spectral function ρ_*s*_(ω) and corresponding Dyson orbitals ϱ_±,*j*_ with relative transition strength. Dyson orbitals have a notably different hierarchy compared to the canonical DFT MOs (Figure 3b). Importantly, neither natural orbitals obtained from the multireference CASCI calculations, see Figure 3e, can explain the experimental STS data, which underlines the importance of the transition probabilities naturally included in the concept of Dyson orbitals. Comparing Figures 4d,e, we find remarkable agreement between the simulated d*I*/d*V* maps derived from Dyson orbitals with the experimental d*I*/d*V* maps, validating the concept of multireference Dyson orbitals to model the ionic resonances observed in STS.

The second example consists of an open-shell Zn^II^Porphyrin molecule (**APor**_**2**_) presented by Sun et al. on Au(111) surface.^36^ The STS measurements reveal a magnetic signal showing the coexistence of Kondo resonance as well as the spin-flip excitation signal at 19 meV. The origin of Kondo resonance, as well as the spatial distribution of d*I*/d*V* maps, was recently rationalized theoretically using the Kondo orbital concept,^37^ considering the triplet ground state obtained from CASCI calculations. Similarly, the spin excitation signal is theoretically rationalized by the triplet-singlet spin excitation.^37^

Here, we will focus on explaining the STS measurements of ionic resonances observed at large bias voltages. As already pointed out in the original work,^36^ canonical DFT orbitals cannot fully explain the experimental STS data. Figure 5a,b shows calculated Frontier canonical orbitals obtained from spin-polarized DFT calculation and corresponding d*I*/d*V* maps. DFT shows ⟨*S* ^2^⟩ value for 2.17 in the ground state. We see that none of the SOMO states labeled A and B can reproduce the experimental d*I*/d*V* map of PIR obtained at −0.4 V, see Figure 5c. Similarly, the lowest NIR resonance acquired at 0.6 V does not match the simulated d*I*/d*V* map of SUMO state labeled C. Note that neither calculated multireference NOs (see Figure 5e) obtained from CASCI calculations cannot reproduce well the experimental d*I*/d*V* maps.

**Figure 5:**
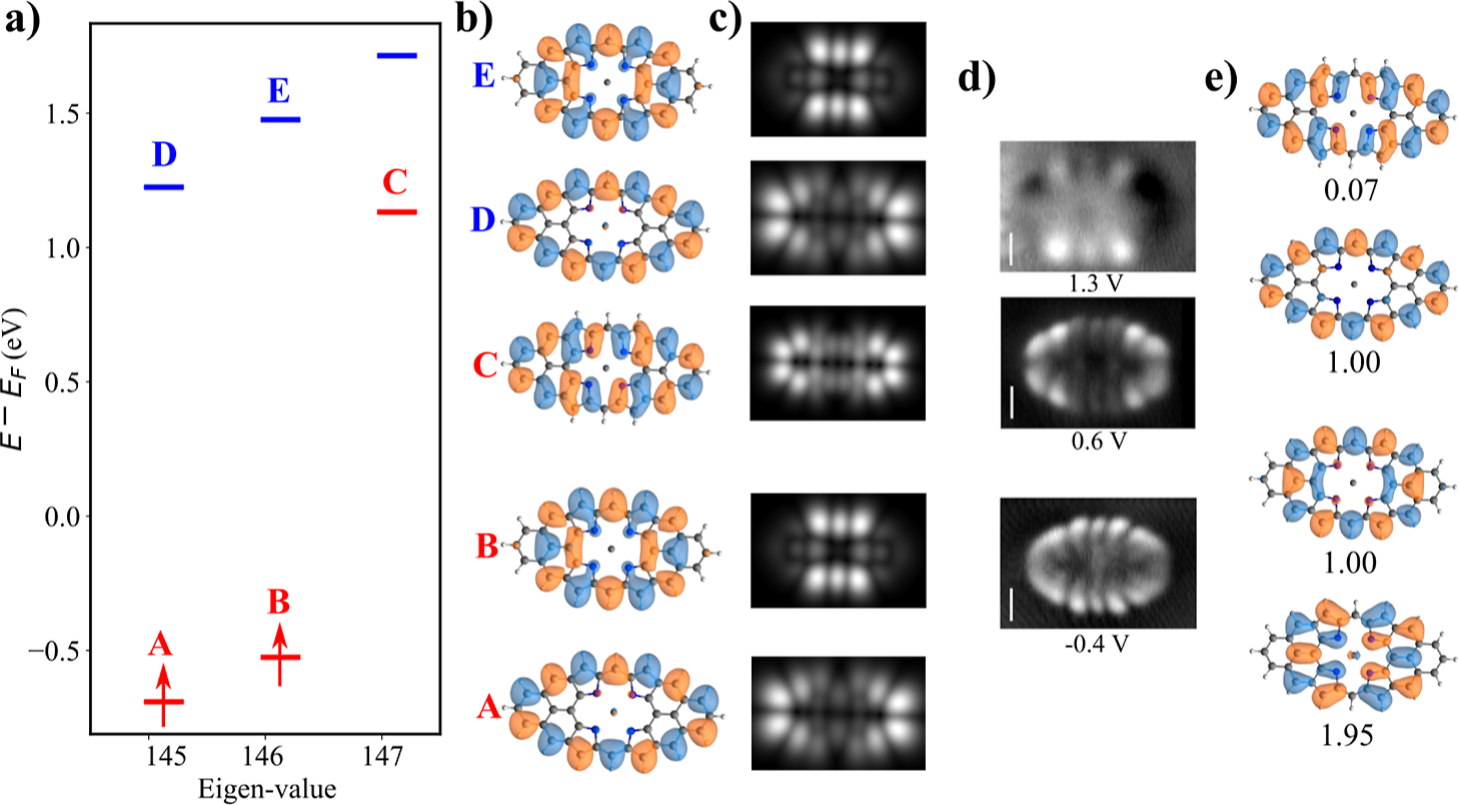
(a) Spin polarized DFT-PBE0 eigen-value; (b) canonical DFT orbitals obtained from spin polarized DFT-PBE0 calculations; (c) simulated d*I*/d*V* maps of corresponding DFT-PBE0 orbitals; (d) experimental d*I*/d*V* maps adapted with permission from ref 36 Copyright 2020 American Chemical Society; (e) multireference natural orbitals obtained from CASCI­(12,12) calculation with corresponding occupation for **APor**_**2**_ molecule.

According to the multireference CASCI­(12,12) calculations performed using the DFT orbitals shown in Figure S4, the ground state wave functions **APor**_**2**_ in the neutral *N* and charged *N* ± 1 states manifest a strong multireference character, as shown in Figure 6a. Therefore, it is essential to transcend the use of the canonical DFT orbitals and employ multireference Dyson orbitals to rationalize the experimental d*I*/d*V* data. In the multireference spectral function ρ_*s*_(ω), shown in Figure 6b, there are two peaks which are very close in energy with a strong overlap. Combination of these two Dyson orbitals provides a very good match to the experimental d*I*/d*V* map of the first PIR acquired at −0.4 V, see Figures 6c–e. Also, the first experimental d*I*/d*V* map of the first NIR agrees with the simulated d*I*/d*V* map corresponding to the Dyson orbital ϱ_+,0_, which is attributed to the transition to the ground *N* + 1 state Ψ_+,0_. The spatial resolution of the second NIR fits well with the Dyson transition ϱ_+,2_ to the second excited *N* + 1 state Ψ_+,2_ instead of the first excited state Ψ_+,1_. Therefore, one would expect to observe first the Dyson transition ϱ_+,1_ instead of ϱ_+,2_. We tentatively attribute this deficiency in theory to the limited size of the active space used in the CASCI­(12,12) calculations, as the energy difference between ϱ_+,1_ and ϱ_+,2_ decreases when increasing the active space.

**Figure 6:**
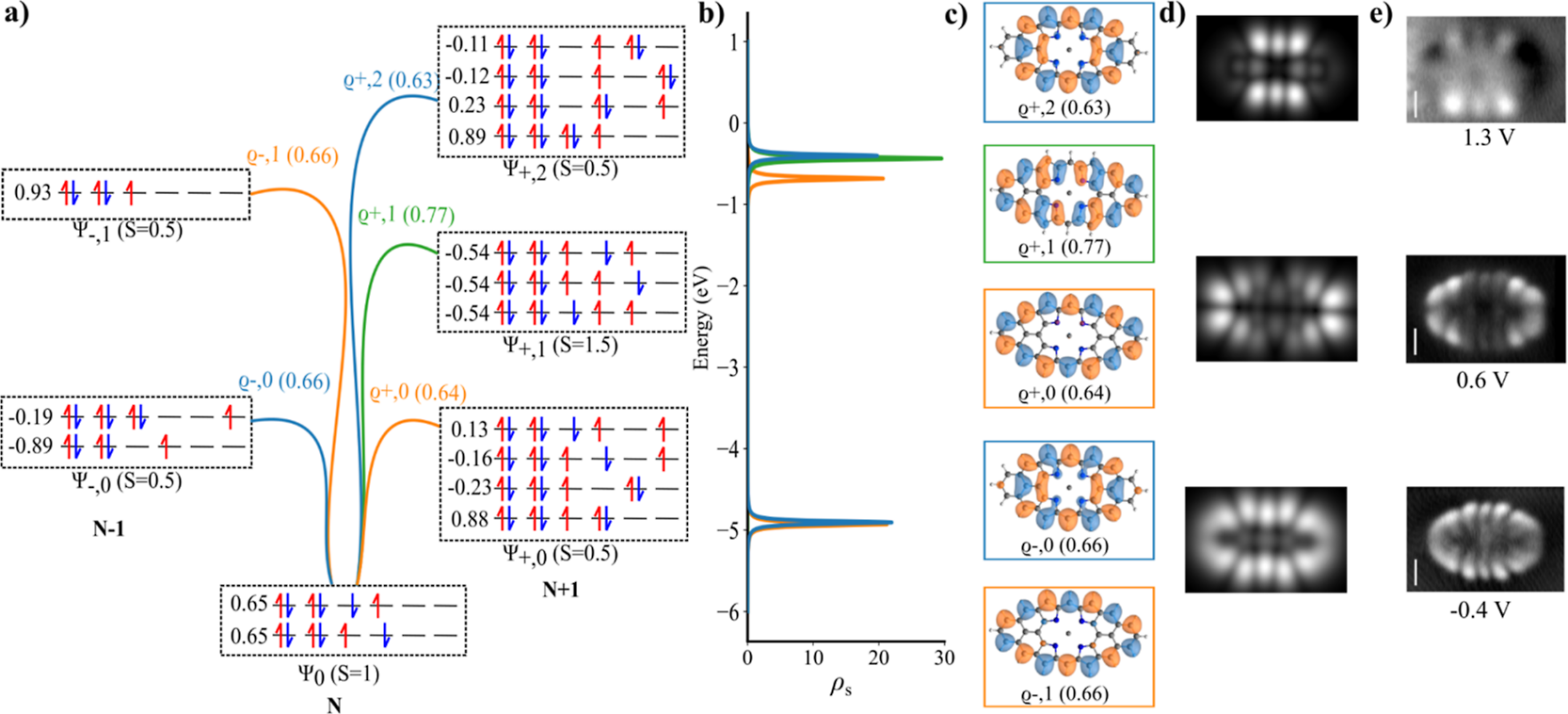
(a) Multireference wave functions of neutral ground state and charge state multiplets; (b) calculated multireference spectral function; (c) multireference Dyson orbitals obtained from CASCI­(11,11) calculations with corresponding strengths; (d) simulated d*I*/d*V* of corresponding Dyson orbitals using PP-STM code^32^ with CO-like tip, and d*I*/d*V* map correspoiding to ϱ_–,0_ and ϱ_–,1_ are added because they are very close in energy from the multireference spectral function; (e) experimental d*I*/d*V* maps adapted with permission from ref 36 Copyright 2025 American Chemical Society for **APor**_**2**_ molecule.

To summarize, the previous discussion clearly showed that STS measurements of strongly correlated molecules cannot provide reliable information about the canonical MOs, nor can they tell us the exact location of the electron before or after removal or addition. What STS measurements track are the transitions, well-represented by the concept of Dyson orbitals. While Dyson orbitals are also referred to as orbitals, they are distinct from the molecular orbitals we typically associate with molecules, as explained in ref^27^. The association of d*I*/d*V* maps with one-electron MOs is problematic to say the least. Therefore, it should be carefully reconsidered when used to discuss general concepts such as the SHI effect, as we will discuss in the next section. The presented framework is not limited to intrinsically open-shell molecules in their neutral state but also extends to systems where open-shell character emerges through charge transfer from a metallic substrate. In such cases, the molecule’s ground-state charge is effectively changed, and the charge-transferred state should be treated as the new reference ground state. Then Dyson orbitals can be computed from this charged ground state by considering *N* ± 1 electron transitions, following the same formalism. This makes the approach broadly applicable to both intrinsic radicals and substrate-induced open-shell systems.

### The SOMO/HOMO Inversion Effect and STS Measurements

Recently, molecules that exhibit the SHI effect have received considerable attention both theoretically and experimentally.^21–24^ These organic radicals feature an electron configuration in which the energy of the SOMO state is presumably below HOMO level. Thus, this concept is based on the assumption of an unusual arrangement of single-electron MOs that contradicts the traditional Aufbau principle of molecules. Recently published STM measurements^25^ reported the presence of the SHI effect for a diradical nonbenzenoid open-shell polycyclic conjugated hydrocarbon (**DNPAH**).

In that work, they found that low bias STM images matches well with the simulated d*I*/d*V* map corresponding to the canonical HOMO orbital obtained from DFT calculations, see Figure 7b–d. Moreover, the spin-unrestricted DFT calculations give the canonical SOMO orbital below HOMO orbital in energy, as shown in Figure 7a and have ⟨*S* ^2^⟩ value of 1.00 in ground state. They rationalized the agreement between the experimental and simulated d*I*/d*V* maps and the reverse energy ordering of the SOMO and HOMO canonical orbitals with respect to the presence of the SHI effect. However, given the previous discussion, there is the question whether STM measurements are in principle suitable to detect the order of MOs in strongly correlated polyradical molecules addressing the SHI effect. Similarly, one can ask what is the meaning of one-electron MOs in case of strongly correlated molecules.

**Figure 7:**
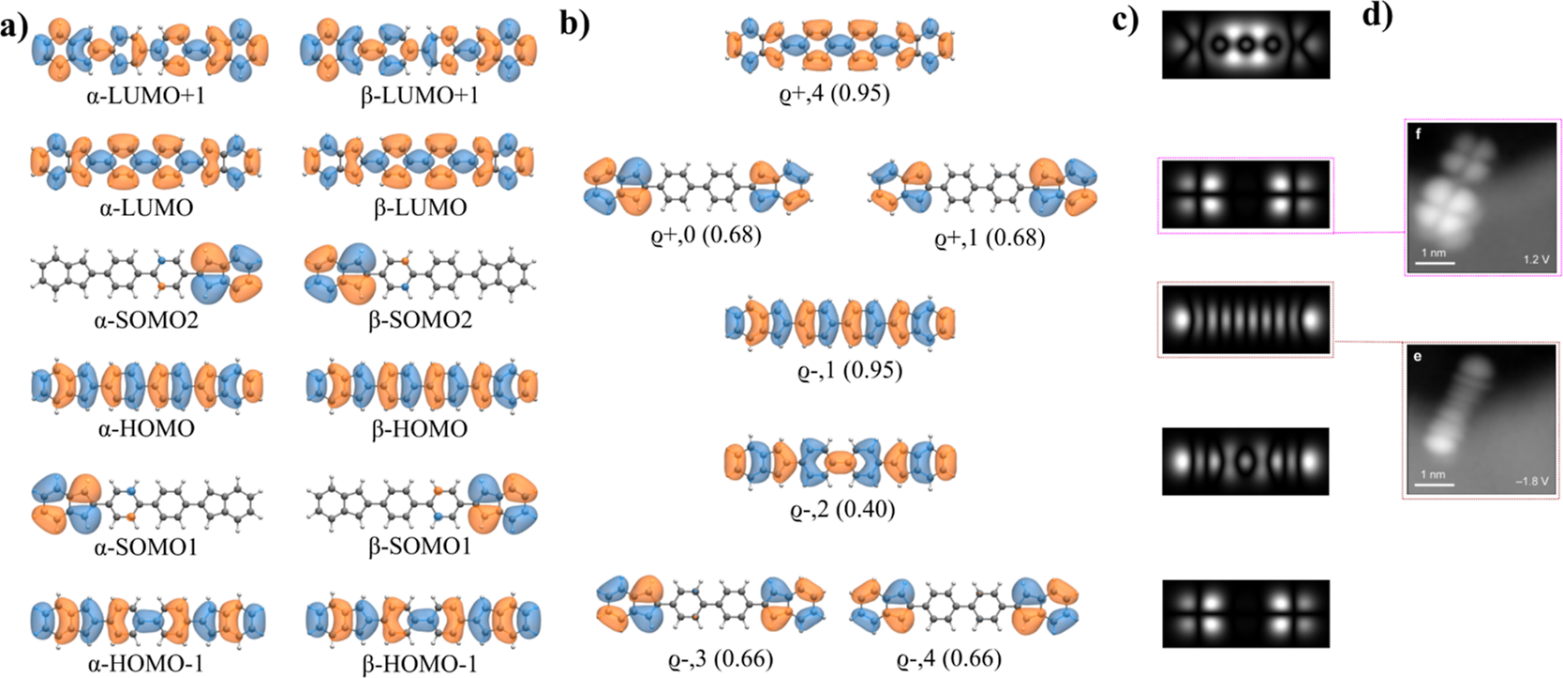
(a) Canonical DFT orbitals obtained from spin polarized DFT-PBE0 calculations; (b) multireference Dyson orbitals obtained from CASCI­(12,12) calculations with corresponding strengths; (c) simulated d*I*/d*V* maps of corresponding Dyson orbitals using PP-STM code^32^ with metal-like tip; (d) experimental constant current topography maps at indicated sample voltages, adapted with permission from ref 25 Copyright 2024 American Chemical Society for **DNPAH** molecule.

To rationalize the STM measurements, we carried out multireference CASCI­(12,12) calculations and Dyson orbitals. The DFT orbitals used for CASCI calculations are shown in Figure S5. Note that the lowest CASCI Dyson orbitals show good agreement with the all-π Dyson orbitals computed using the DMRG method, as presented in Figure S7. The CASCI calculation determines the diradical singlet ground *N* state Ψ_0_ with two SONO orbitals with occupancy 1. Figure 8a shows possible transitions from the neutral ground state Ψ_0_ to the low-energy charged Ψ_±,*j*_ states, including their transition strengths. We observe that two transitions from the singlet (*S* = 0) ground state Ψ_0_ to high spin charged states (*S* = 1.5) Ψ_–,0_ and Ψ_+,3_, are not allowed due to the spin conservation. This can be understood by the fact that (de)­attachment of a single electron cannot convert the ground Ψ_0_ state to those high spin Ψ_+,3_ and Ψ_+,3_ states, see Figure 8a. Figure 8b shows the resulting spectral function with the allowed Dyson orbitals ϱ_±,*j*_. We can see that the simulated d*I*/d*V* map of the Dyson orbital ϱ_–,1_ matches nicely to the experimental STM maps attributed to the lowest PIR. We can see, that the Dyson ϱ_–,1_ orbital corresponds to the process, where an electron is detached mostly from HOMO orbital. This explains why simulated d*I*/d*V* maps obtained from the canonical HOMO orbital can approximate the experimental data. Similarly, the first NIR corresponds to an electron attachment causing the transition from the neutral ground Ψ_0_ state to doubly degenerate Ψ_+,0_ and Ψ_+,1_ states. This process is represented by two similar Dyson orbitals ϱ_+,0_ and ϱ_+,1_, see Figure 8b. The simulated d*I*/d*V* maps, see Figure 7c, reproduce nicely the experimental STM maps in Figure 7d. Here, the attachment process cannot be easily associated with single MOs, as the states have a strong multireference character.

**Figure 8:**
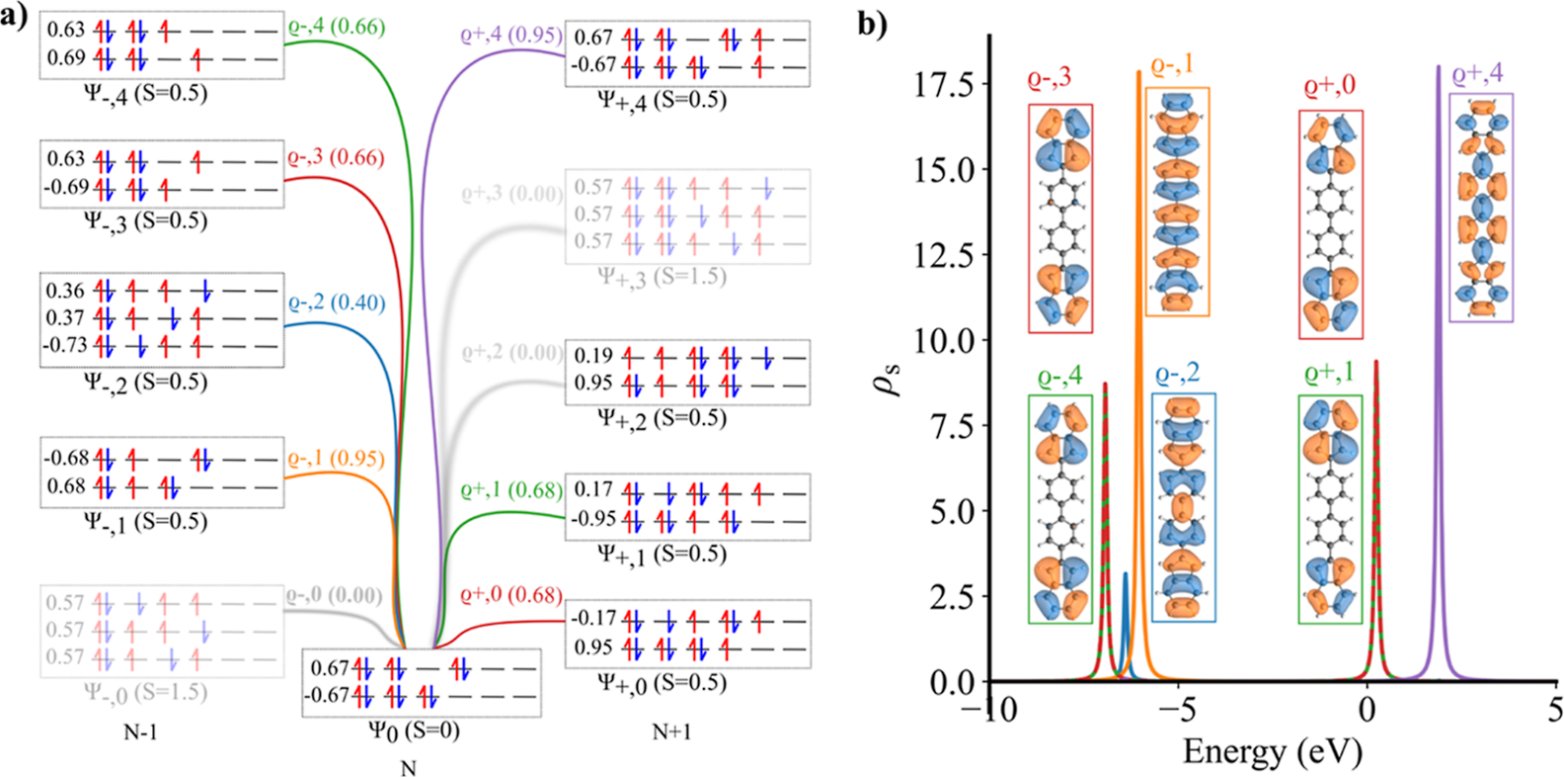
(a) Multireference wave functions of neutral ground state and charge state multiplet (b) multireference spectral function for the **DNPAH** molecule.

From the discussion, it is evident that in particular transitions from the ground *N* state to charged *N* ± 1 states due to the electron tunneling processes can significantly affect the resulting contrast of the d*I*/d*V* map. Therefore, the interpretation of STS measurements of polyradical molecules based on association with one-electron canonical MOs is problematic.

The primary source of confusion arises from comparing one-electron DFT canonical orbitals with STM/STS maps, where the energy ordering and orbital maps appear to align well with experimental observations. In a closed-shell molecule, we can define a unique energy ordering, often referred to as single-electron energy. However, for open-shell molecules, this concept loses its relevance, as a single energy cannot be unambiguously assigned to a specific MO. As illustrated in Figure 2a,b, the spectral function exhibits distinct energy levels corresponding to each Dyson orbital, making it seemingly possible to assign an energy to these orbitals. However, for open-shell molecules, as shown in Figure 8b, the SOMO and SUMO do not have well-defined energy localization. Instead, their energies can be found both above the highest occupied molecular orbital (HOMO) and below another lower-energy orbital. This inconsistency makes it fundamentally contradictory to assign a single energy value to molecular orbitals derived from Dyson orbitals.

Moreover, for the open-shell singlet ground state, the correct wave function is a linear combination of two Slater determinants corresponding to the configurations |↑↓⟩ + |↓↑⟩, see Figure 8a. Single-determinant approaches, such as DFT method, inherently fail to capture this multireference character. Instead, they adopt one of the configurations, either |↑↓⟩ or |↓↑⟩, leading to a significant deviation from the actual physical state. To compensate for this, single-reference methods introduce an artificial spatial splitting of the Frontier HOMO and LUMO orbitals, resulting in what is termed a *broken-spin symmetry solution*. However, this solution lacks any physical meaning, especially regarding the energy ordering of canonical MOs. This artifact is particularly relevant for the correct interpretation of the SHI effect. Here, the energies of SOMO and HOMO states are simply a consequence of the inadequacies of the unrestricted DFT method rather than a genuine feature of the electronic structure. More rigorous analysis of the description of the open-shell singlet state is provided in Supporting Information.

The second problem is associated with the STS measurement itself, because they include transitions associated with the (de)­attachment of electrons. Therefore, the STS/STM measurement itself influences the ground state Ψ_0_ of the molecule. Moreover, certain electronic transitions are inherently forbidden due to selection rules. As a result, STS spectra only capture allowed electronic transitions, meaning that STS measurements provide incomplete information about the electronic structure and energy ordering of molecular orbitals. The STS measurements provide information about the ionization resonances of molecules, which, especially in the case of open-shell molecules, can differ significantly from the MO picture. Thus, STM lacks the ability to assign energy ordering to single-electron molecular orbitals. Importantly, STM does not directly observe molecular orbitals or their intrinsic energy ordering, as previously highlighted in ref 7 STM measurements do not provide direct information about the electronic structure of a neutral molecule. Instead, they perturb the system and detect electronic transitions, which inherently do not reveal the molecular orbitals of the neutral state or their corresponding energy levels.

If one constructs Dyson orbitals for **DNPAH** molecule based on closed-shell DFT orbitals, where the HOMO is lower in energy than the singly occupied state, the resulting Dyson orbital ordering remains consistent. This demonstrates that Dyson orbitals do not directly reflect the single-electron energies of molecular orbitals, but rather the transitions observed in STM.

## Conclusions

In conclusion, we presented the concept of multireference Dyson orbitals, which provides a reliable methodology to rationalize STS/STM measurements. Moreover, the concept of Dyson orbitals also avoids controversy regarding the observation of MOs in STS experiments, which violates the basic principles of quantum mechanics. It enables us to incorporate two important ingredients: (i) the electronic transitions from the neutral ground state to charged low-energy states invoked by the single electron tunneling; (ii) describing multireference electronic states of strongly correlated molecules. We showed that the concept of one-electron MOs can approximate STS spectra of closed-shell molecules, while it provides a completely misleading picture in the case of strongly correlated molecules. Therefore, the multireference Dyson orbitals are mandatory to rationalize STS measurements of strongly correlated polyradical molecules. We also demonstrate that estimating the energy order of single-electron MOs from STS measurements of strongly correlated open-shell molecules is problematic.

In this context, STS measurements for strongly correlated molecules cannot provide any information about the energies of one-electron MOs. Therefore, STS measurements cannot address SOMO-HOMO inversion, which concept is inherently associated with one-electron approximation.

Due to the ambiguity between single-electron MOs and STS measurements for the strongly correlated polyradical molecules, we recommend labeling individual resonances as positive/negative ionic resonances instead of the commonly adopted notation using canonical MOs, e.g. HOMO/LUMO, etc. We believe that changing the nomenclature will help to improve the somewhat short-sighted interpretations of STS/STM measurements.

## Methods

The geometries of all molecules were optimized in their respective ground states using density functional theory (DFT) as implemented in the FHI-AIMS software package.^38^ The hybrid PBE0 functional^35^ was employed for these calculations, with the Tkatchenko-Scheffler^39^ method incorporated to account for van der Waals interactions. To accurately capture the electronic structure, Complete Active Space Configuration Interaction (CASCI) calculations were performed. The one- and two-body integrals were generated using the ORCA quantum chemistry software,^40^ based on the closed-shell DFT orbitals obtained with the PBE0 ^35^ exchange–correlation functional. For the pentacene, **APor**_**2**_, and **DNPAH** molecules, an active space of CASCI­(12,12) was employed. Due to the odd number of electrons in the **TTAT** molecule, the restricted open-shell Kohn–Sham (ROKS) approach was used, with an active space of CASCI­(11,11). After constructing the full many-body Hamiltonian from these integrals, we diagonalized the Hamiltonian and constructed Dyson orbitals using our in-house many-body code. The simulated d*I*/d*V* maps of the Dyson orbitals were calculated using the Probe Particle Scanning Probe Microscopy (PP-SPM) code.^32^

## Supplementary Material



## Notes

It includes a demonstration of the non-uniqueness of orbitals, a detailed derivation of the wave function for a system of two electrons in two orbitals, and a derivation outlining the recovery of molecular orbitals from Dyson orbitals in cases where the system can be described within the framework of DFT; Furthermore, it contains the DFT orbitals used in CASCI calculations, the wave function representation of the TTAT molecule in different *S*_*z*_ subspaces, and the DMRG-computed Dyson orbitals of the diradical DNPAH (PDF)

## References

[1] Binnig G., Rohrer H., Gerber C., Weibel E. (1982). Surface Studies by
Scanning Tunneling Microscopy. Phys. Rev. Lett..

[2] Chen, C. J. Introduction to Scanning Tunneling Microscopy; Oxford University Press Oxford, 2007; . 10.1093/acprof:oso/9780199211500.001.0001.

[3] Stroscio J. A., Feenstra R. M., Fein A. P. (1986). Electronic
Structure of the Si(111)­2
× 1 Surface by Scanning-Tunneling Microscopy. Phys. Rev. Lett..

[4] Zandvliet H. J., van Houselt A. (2009). Scanning Tunneling Spectroscopy. Annu. Rev. Anal. Chem..

[5] Chiang S. (1997). Scanning Tunneling
Microscopy Imaging of Small Adsorbed Molecules on Metal Surfaces in
an Ultrahigh Vacuum Environment. Chem. Rev..

[6] Repp J., Meyer G., Stojković S. M., Gourdon A., Joachim C. (2005). Molecules
on Insulating Films: Scanning-Tunneling Microscopy Imaging of Individual
Molecular Orbitals. Phys. Rev. Lett..

[7] Pham B. Q., Gordon M. S. (2017). Can orbitals really be observed in
scanning tunneling
microscopy experiments?. J. Phys. Chem. A.

[8] Krylov A. I. (2020). From orbitals
to observables and back. J. Chem. Phys..

[9] Scerri E. R. (2000). Have Orbitals
Really Been Observed?. J. Chem. Educ..

[10] Zhao Y., Jiang K., Li C., Liu Y., Zhu G., Pizzochero M., Kaxiras E., Guan D., Li Y., Zheng H., Liu C., Jia J., Qin M., Zhuang X., Wang S. (2023). Quantum nanomagnets in on-surface
metal-free porphyrin chains. Nat. Chem..

[11] Li J., Sanz S., Castro-Esteban J., Vilas-Varela M., Friedrich N., Frederiksen T., Peña D., Pascual J. I. (2020). Uncovering the Triplet Ground State
of Triangular Graphene
Nanoflakes Engineered with Atomic Precision on a Metal Surface. Phys. Rev. Lett..

[12] Pavlicek N., Mistry A., Majzik Z., Moll N., Meyer G., Fox D. J., Gross L. (2017). Synthesis
and characterization of
triangulene. Nat. Nanotechnol..

[13] Su J., Lyu P., Lu J. (2023). Atomically precise imprinting π-magnetism in
nanographenes via probe chemistry. Precis. chem..

[14] Song S. (2024). Highly entangled polyradical nanographene with coexisting
strong
correlation and topological frustration. Nat.
Chem..

[15] Zuzak R., Kumar M., Stoica O., Soler-Polo D., Brabec J., Pernal K., Veis L., Blieck R., Echavarren A. M., Jelinek P. (2024). On-Surface
Synthesis
and Determination of the Open-Shell Singlet Ground State of Tridecacene. Angew. Chem., Int. Ed..

[16] Villalobos F. (2025). Globally aromatic odd-electron π-magnetic macrocycle. Chem..

[17] Calupitan J. P., Berdonces-Layunta A., Aguilar-Galindo F., Vilas-Varela M., Peña D., Casanova D., Corso M., de Oteyza D. G., Wang T. (2023). Emergence of π-Magnetism in Fused Aza-Triangulenes: Symmetry
and Charge Transfer Effects. Nano Lett..

[18] Vilas-Varela M., Romero-Lara F., Vegliante A., Calupitan J. P., Martínez A., Meyer L., Uriarte-Amiano U., Friedrich N., Wang D., Schulz F. (2023). On-Surface
Synthesis and Characterization of a High-Spin Aza-[5]-Triangulene. Angew. Chem..

[19] Toroz D., Rontani M., Corni S. (2013). Proposed Alteration of Images of
Molecular Orbitals Obtained Using a Scanning Tunneling Microscope
as a Probe of Electron Correlation. Phys. Rev.
Lett..

[20] Schulz F., Ijäs M., Drost R., Hämäläinen S. K., Harju A., Seitsonen A. P., Liljeroth P. (2015). Many-body
transitions in a single molecule visualized by scanning tunnelling
microscopy. Nat. Phys..

[21] Kasemthaveechok S., Abella L., Crassous J., Autschbach J., Favereau L. (2022). Organic radicals with inversion of SOMO and HOMO energies
and potential applications in optoelectronics. Chem. Sci..

[22] Kumar A., Sevilla M. D. (2018). SOMO–HOMO
Level Inversion in Biologically Important
Radicals. J. Phys. Chem. B.

[23] Westcott B. L., Gruhn N. E., Michelsen L. J., Lichtenberger D. L. (2000). Experimental
Observation of Non-Aufbau Behavior: Photoelectron Spectra of Vanadyloctaethylporphyrinate
and Vanadylphthalocyanine. J. Am. Chem. Soc..

[24] Kusamoto T., Kume S., Nishihara H. (2008). Realization of SOMO- HOMO level conversion
for a TEMPO-dithiolate ligand by coordination to platinum (II). J. Am. Chem. Soc..

[25] Mishra S., Vilas-Varela M., Fatayer S., Albrecht F., Peña D., Gross L. (2024). Observation of SOMO-HOMO Inversion in a Neutral Polycyclic Conjugated
Hydrocarbon. ACS Nano.

[26] Yu P., Kocić N., Repp J., Siegert B., Donarini A. (2017). Apparent reversal
of molecular orbitals reveals entanglement. Phys. Rev. Lett..

[27] Truhlar D. G., Hiberty P. C., Shaik S., Gordon M. S., Danovich D. (2019). Orbitals and
the interpretation of photoelectron spectroscopy and (e, 2e) ionization
experiments. Angew. Chem..

[28] Ortiz J. (2020). Dyson-orbital
concepts for description of electrons in molecules. J. Chem. Phys..

[29] Kohn W., Sham L. J. (1965). Self-Consistent
Equations Including Exchange and Correlation
Effects. Phys. Rev..

[30] Szabo, A. ; Ostlund, N. S. Modern Quantum Chemistry: Introduction to Advanced Electronic Structure Theory; Revised Edition; Dover Publications: New York, 1989.

[31] Eriksen J. J., Baudin P., Ettenhuber P., Kristensen K., Kjærgaard T., Jørgensen P. (2015). Linear-Scaling Coupled Cluster with
Perturbative Triple Excitations: The Divide–Expand–Consolidate
CCSD­(T) Model. J. Chem. Theory Comput..

[32] Krejčí O., Hapala P., Ondráček M., Jelínek P. (2017). Principles
and simulations of high-resolution STM imaging with a flexible tip
apex. Phys. Rev. B.

[33] Tersoff J., Hamann D. R. (1985). Theory of the scanning tunneling microscope. Phys. Rev. B.

[34] Vegliante A., Vilas-Varela M., Ortiz R., Romero Lara F., Kumar M., Gómez-Rodrigo L., Trivini S., Schulz F., Soler-Polo D., Ahmoum H. (2025). On-Surface
Synthesis of a Ferromagnetic Molecular Spin Trimer. J. Am. Chem. Soc..

[35] Adamo C., Barone V. (1999). Toward reliable density functional methods without
adjustable parameters: The PBE0 model. J. Chem.
Phys..

[36] Sun Q., Mateo L. M., Robles R., Ruffieux P., Lorente N., Bottari G., Torres T., Fasel R. (2020). Inducing open-shell
character in porphyrins through surface-assisted phenalenyl π-extension. J. Am. Chem. Soc..

[37] Calvo-Fernández A., Kumar M., Soler-Polo D., Eiguren A., Blanco-Rey M., Jelínek P. (2024). Theoretical model for multiorbital Kondo screening
in strongly correlated molecules with several unpaired electrons. Phys. Rev. B.

[38] Blum V., Gehrke R., Hanke F., Havu P., Havu V., Ren X., Reuter K., Scheffler M. (2009). Ab initio Mol. Simul.s with numeric
atom-centered orbitals. Comput. Phys. Commun..

[39] Tkatchenko A., Scheffler M. (2009). Accurate Molecular
Van Der Waals Interactions from
Ground-State Electron Density and Free-Atom Reference Data. Phys. Rev. Lett..

[40] Neese F. (2012). The ORCA program
system. Wiley Interdiscip. Rev.: Comput. Mol.
Sci..

