# Supplementary material for: Multireference Theory of Scanning Tunneling Spectroscopy Beyond One-Electron Molecular Orbitals: Can We Image Molecular Orbitals?
Source: J Am Chem Soc. 2025 Jul 2;147(28):24993–5003. doi: 10.1021/jacs.5c08166 (PMC12272701; doi:10.1021/jacs.5c08166)
Supplement: Supplementary file 1 [file ja5c08166_si_001.pdf]

Supporting Information: Multireference theory of scanning tunneling spectroscopy beyond one-electron molecular orbitals: can we image molecular orbitals?

Manish Kumar,[*,†,‡] Diego Soler-Polo,[*,†] Marco Lozano,[†] Enzo Monino,[¶] Libor Veis,[¶] and Pavel Jelínek[*,†,§]

†*Institute of Physics, Czech Academy of Sciences, Prague 16200, Czech Republic*

‡*Department of Condensed Matter Physics, Faculty of Mathematics and Physics, Charles University, CZ12116 Prague 2, Czech Republic*

¶*Department of Theoretical Chemistry, J. Heyrovsky Institute of Physical Chemistry, Czech Academy of Sciences, Prague 18200, Czech Republic*

§*Czech Advanced Technology and Research Institute (CATRIN), Palacký University Olomouc, 779 00 Olomouc, Czech Republic*

E-mail: kumarm@fzu.cz; soler@fzu.cz; jelinekp@fzu.cz

Contents

1 Theoretical Details **S-2**

2 Non Uniqueness of Orbitals **S-3**

3 Two electron in two orbitals **S-4**

4 Recovering molecular orbitals S-7

5 DFT Orbitals used for CASCI S-9

6 Wavefunction Representation of the TTAT Molecule in Different S_z subspace S-11

7 DMRG Dyson Orbitals of DNPAH diradical S-13

References S-14

1 Theoretical Details

The geometries of all molecules were optimized in their respective ground states using density functional theory (DFT) as implemented in the FHI-AIMS software package.[S1] The hybrid PBE0 functional[S2] was employed for these calculations, with the Tkatchenko-Scheffler[S3] method incorporated to account for van der Waals interactions. To accurately capture the electronic structure, Complete Active Space Configuration Interaction (CASCI) calculations were performed. The one- and two-body integrals were generated using the ORCA quantum chemistry software,[S4] based on the closed-shell DFT orbitals obtained with the PBE0[S2] exchange-correlation functional. For the pentacene, **APor$_2$**, and **DNPAH** molecules, an active space of CASCI(12,12) was employed. Due to the odd number of electrons in the **TTAT** molecule, the restricted open-shell Kohn-Sham (ROKS) approach was used, with an active space of CASCI(11,11). After constructing the full many-body Hamiltonian from these integrals, we diagonalized the Hamiltonian and constructed Dyson orbitals using our in-house many-body code. The simulated dI/dV maps of the Dyson orbitals were calculated using the Probe Particle Scanning Probe Microscopy (PP-SPM) code[S5] for a CO-like tip.

2 Non Uniqueness of Orbitals

Besides the fact that molecular orbitals cannot be directly associated with any observable quantities, and they can be defined in multiple ways and STM does not provide information about the specific orbitals from which electrons are removed or where they go when added. Instead, STM offers spatial maps that capture transitions between the N and $N \pm 1$ states, which are described by Dyson orbitals. As illustrated in Figure S1, we can construct a many-body wavefunction using various types of orbitals like DFT orbitals, localized orbitals for example. The many-body natural orbitals and Dyson orbitals are uniquely defined, regardless of the specific orbitals chosen for the construction. In this sense, STM does not provide information about the molecular orbitals nor can it tell us the exact location of the electron before or after removal or addition. What STM can track are the transitions, which are well represented by Dyson orbitals. Although Dyson orbitals are also called orbitals, they are different from the molecular orbitals that we typically associate with molecules, as explained in.[S6]

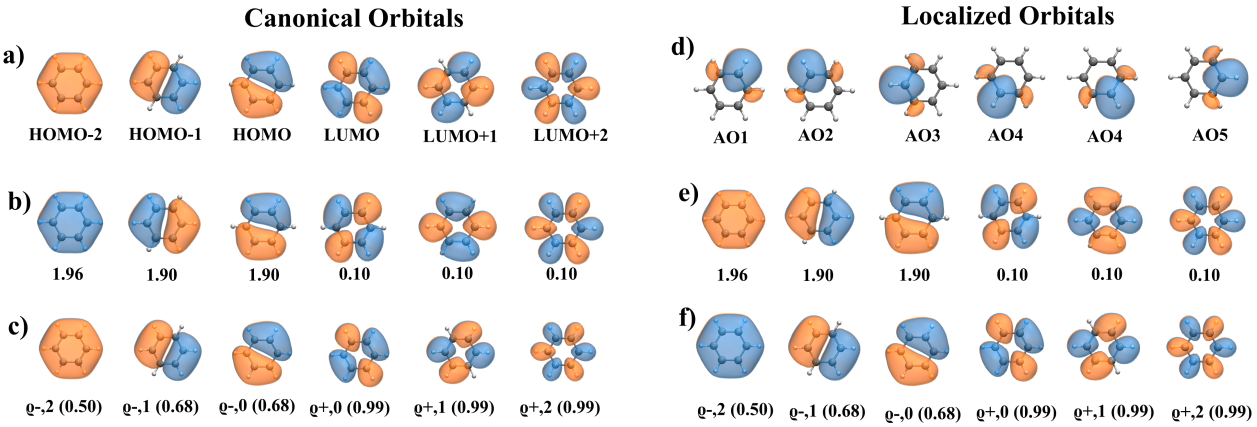

Figure S1: a) DFT orbitals b) many-body natural orbitals with the occupation below constructed by starting from the DFT orbitals. c) many-body Dyson orbitals constructed by starting from DFT orbitals. d) Local orbitals of π space . e) many-body natural orbitals with occupation below constructed by starting from the the localized orbitals. f) many-body dyson orbitals constructed by starting from localized orbitals.

3 Two electron in two orbitals

Wavefunction Analysis of Diradical System in DFT Calculations

Considering two electrons in two orbitals we can analyze the wavefunctions associated with diradical systems in Density Functional Theory (DFT) calculations. Here, we examine cases where the triplet and singlet configurations are the ground states, demonstrating the limitations of DFT in handling open-shell singlet states, especially when broken-symmetry solutions arise.

Triplet State with $M_S = 1$

Consider the case where we have two electrons with both spins up. The Slater determinant for the $M_S = +1$ triplet state can be expressed with two electrons in different orbitals as follows:

$$\Psi_{S=1,M_S=+1} = \frac{1}{\sqrt{2}} \begin{vmatrix} \varphi_A(1)\alpha(1) & \varphi_A(2)\alpha(2) \\ \varphi_B(1)\alpha(1) & \varphi_B(2)\alpha(2) \end{vmatrix}$$

Upon simplification:

$$\Psi_{S=1,M_S=+1} = \frac{1}{\sqrt{2}} \left(\varphi_A(1)\alpha(1)\varphi_B(2)\alpha(2) - \varphi_A(2)\alpha(2)\varphi_B(1)\alpha(1)\right)$$

Separating spin and spatial components, we get:

$$\Psi_{S=1,M_S=+1} = \frac{1}{\sqrt{2}} \left(\varphi_A(1)\varphi_B(2) - \varphi_A(2)\varphi_B(1)\right)\alpha(1)\alpha(2)$$

This wavefunction is antisymmetric in its total form, with the spatial part being antisymmetric and the spin part symmetric. It is an eigenfunction of the $\hat{S}^2$ operator:

$$\hat{S}^2\Psi_{S=1} = S(S+1)\Psi_{S=1} = 2\Psi_{S=1}$$

Triplet State with $M_S = -1$

For the $M_S = -1$ triplet state, the wavefunction is obtained by substituting α with β:

$$\Psi_{S=1,M_S=-1} = \frac{1}{\sqrt{2}} \left(\varphi_A(1)\varphi_B(2) - \varphi_A(2)\varphi_B(1)\right) \beta(1)\beta(2)$$

This state is also an eigenfunction of the $\hat{S}^2$ operator with the same eigenvalue.

Singlet State with $M_S = 0$

In the singlet $M_S = 0$ state, one electron is spin-up and the other is spin-down. The wavefunction, in determinant form, is:

$$\Psi_{M_S=0} = \frac{1}{\sqrt{2}} \begin{vmatrix} \varphi_A(1)\alpha(1) & \varphi_A(2)\beta(2) \\ \varphi_A(1)\beta(1) & \varphi_A(2)\alpha(2) \end{vmatrix}$$

After simplification, we have:

$$\Psi_{M_S=0} = \frac{1}{\sqrt{2}} \left(\varphi_A(1)\alpha(1)\varphi_A(2)\beta(2) - \varphi_A(2)\alpha(2)\varphi_A(1)\beta(1)\right)$$

This is a closed-shell singlet solution, with a symmetric spatial part and an antisymmetric spin part. It is also an eigenfunction of $\hat{S}^2$ with eigenvalue zero:

$$\hat{S}^2\Psi_{S=0} = S(S+1)\Psi_{S=0} = 0$$

Triplet State with $M_S = 0$: Broken-Symmetry Solution

In the $M_S = 0$ triplet state, both electrons are in different orbitals with opposite spins. The broken-symmetry solutions for this state are as follows:

$$\Psi_{M_S=0} = \frac{1}{\sqrt{2}} \left(\varphi_A(1)\varphi_B(2)\alpha(1)\beta(2) - \varphi_A(2)\varphi_B(1)\alpha(2)\beta(1)\right)$$
$$\Psi_{M_S=0} = \frac{1}{\sqrt{2}} \left(\varphi_A(1)\varphi_B(2)\beta(1)\alpha(2) - \varphi_A(2)\varphi_B(1)\beta(2)\alpha(1)\right)$$

Each broken-symmetry solution is not an eigenfunction of $\hat{S}^2$ and does not represent a

pure spin state, resulting in spin contamination.

Physical Interpretation of Broken-Symmetry Solutions

In DFT, open-shell singlet solutions are approximated using broken-symmetry solutions, which are not eigenfunctions of $\hat{S}^2$. If we combine both broken-symmetry solutions, we obtain:

$$\Psi_{S=1,M_S=0} = \frac{1}{2}\left(\varphi_A(1)\varphi_B(2) - \varphi_A(2)\varphi_B(1)\right)\left(\alpha(1)\beta(2) + \beta(1)\alpha(2)\right)$$

This is the triplet state with $M_S = 0$. By contrast, if we subtract these solutions, we get the singlet state:

$$\Psi_{S=0,M_S=0} = \frac{1}{2}\left(\varphi_A(1)\varphi_B(2) + \varphi_A(2)\varphi_B(1)\right)\left(\alpha(1)\beta(2) - \beta(1)\alpha(2)\right)$$

Here, the spatial part is symmetric, and the spin part is antisymmetric, representing the singlet state.

Checking for Broken-Symmetry Solutions

To verify if a DFT solution is a broken-symmetry solution, we calculate $\langle S^2 \rangle$ as[S7] :

$$\langle S^2 \rangle_{MFH} = \langle S^2 \rangle_{exact} + N_\beta - \sum_{ij}^{occ} |\langle \psi_i^\alpha | \psi_j^\beta \rangle|^2$$

where:

$$\langle S^2 \rangle_{exact} = \left(\frac{N_\alpha - N_\beta}{2}\right)\left(\frac{N_\alpha - N_\beta}{2} + 1\right)$$

Broken-symmetry solutions are commonly used in DFT but lack pure spin character due to spin contamination, impacting their utility in describing accurate electronic structure.

4 Recovering molecular orbitals

In this section, we show explicitly how in the limit where a one-electron approximation holds, the Dyson orbitals are reproduced by the canonical orbitals. Let us consider two states, $|\Psi_0\rangle$, $|\Psi_+\rangle$, respectively for the neutral state (with N electrons) and the charged system ($N+1$ electrons).

Let us assume these states are well described by Slater determinants, as is the case in one-electron methods like DFT:

$$|\Psi_+\rangle = \mathrm{Det}\left(\phi_1, \ldots, \phi_{N+1}\right) = \frac{1}{\sqrt{(N+1)!}} \sum_{\sigma \in S_{N+1}} (-1)^{|\sigma|} \prod_{j=1}^{N+1} \phi_{\sigma(j)}(x_j)$$

$$|\Psi_0\rangle = \mathrm{Det}\left(\varphi_1, \ldots, \varphi_N\right) = \frac{1}{\sqrt{N!}} \sum_{\tau \in S_N} (-1)^{|\tau|} \prod_{j=1}^{N} \varphi_{\tau(j)}(x_j),$$

where in general we have different spin-orbitals $\{\phi\}$ and $\{\varphi\}$ (which do not need to be mutually orthogonal) to describe the two wavefunctions.

The Dyson orbital corresponding to these two states is given by the multireference overlap:

$$\varphi_+(x_{N+1}) = \sqrt{N+1} \int \mathrm{Det}\left(\phi_1, \ldots, \phi_{N+1}\right) \mathrm{Det}\left(\varphi_1, \ldots, \varphi_N\right) dx_1 \ldots dx_N.$$

Now let us expand the spin-orbitals in a common basis, $\{\chi_\mu(x)\}$, which we can think of as the basis set of our one-electron calculation:

$$\phi_a(x) = \sum_\mu \phi_{a\mu} \chi_\mu(x)$$

and

$$\varphi_b(x) = \sum_\nu \varphi_{b\nu} \chi_\nu(x).$$

Such expansion is the result of performing DFT or any other one-electron method for the neutral and charged system.

Let us further define the overlap matrix $\boldsymbol{D}$ with elements $D_{a,b}$:

$$D_{a,b} = \int \phi_a(x)\varphi_b(x)dx.$$

Notice that if the orbitals for the neutral and charged system are the same (as is usually assumed), the matrix $\boldsymbol{D}$ is the identity.

In general, however, by defining the basis set overlap $S_{\mu\nu} = \int \chi_\mu(x)\chi_\nu(x)dx$, we see that we can compactly write:

$$\boldsymbol{D} = \boldsymbol{\phi}\boldsymbol{S}\boldsymbol{\varphi}^t$$

.

Introducing further the notation

$$d(\sigma) = \frac{(-1)^{|\sigma|}}{N!}\sum_{\tau\in S_N}(-1)^{|\tau|}\prod_{j=1}^{N} D_{\sigma(j)\tau(j)},$$

a straight-forward manipulation yields the expression:

$$\varphi_+(x) = \sum_{\sigma\in S_{N+1}} d(\sigma)\phi_{\sigma(N+1)}(x).$$

The matrix $\boldsymbol{D}$ will typically be close to the identity. In most practical calculations, the shape of the orbitals is not reoptimized for the charged system. In such cases, $\boldsymbol{D}$ is *exactly* the identity, since $d(\sigma)$ is 1 if $\sigma(N+1) = N+1$ and $d(\sigma) = 0$ otherwise, because in this case $D_{\sigma(j)\tau(j)} = \delta_{\sigma(j)\tau(j)}$.

In such a case is then clear that:

$$\varphi_+(x) = \phi_{N+1}(x),$$

so that the Dyson orbital coincides exactly with the canonical molecular orbital.

This simple calculation shows how the molecular orbitals serve as a first approximation to the multireference transitions. However, even without treating the correlation beyond the one-electron picture, we see how just a calculation of molecular orbitals for a neutral system will fail to yield the correct ordering of the observed transitions, since it also does not account in general for the re-optimization of the electronic structure for different charged states.

5 DFT Orbitals used for CASCI

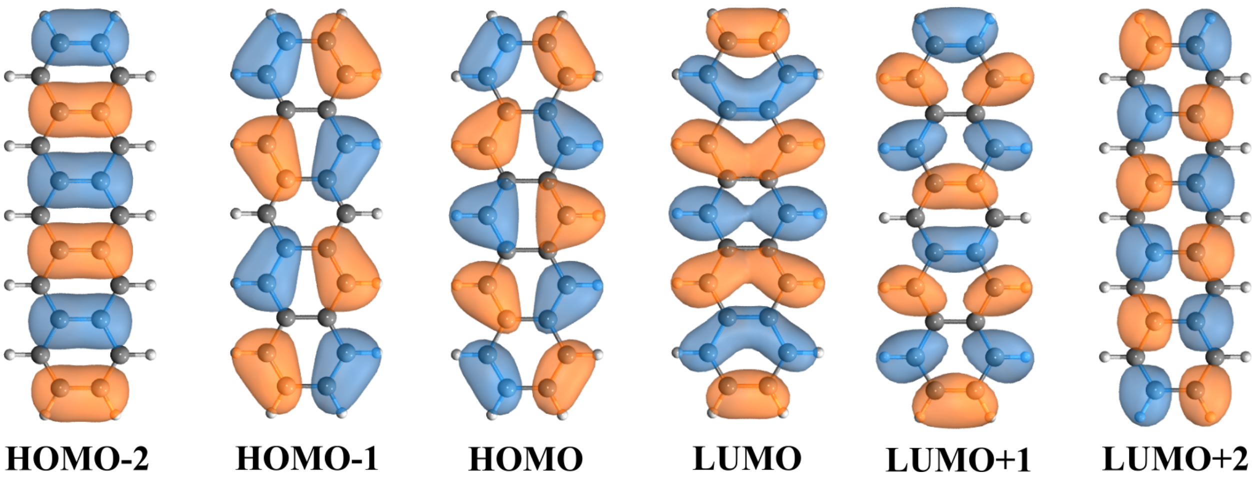

Figure S2: Frontier orbitals around the Fermi level used in CAS(12,12) obtained from the spin-unpolarized DFT-PBE0 calculation for the pentacene molecule.

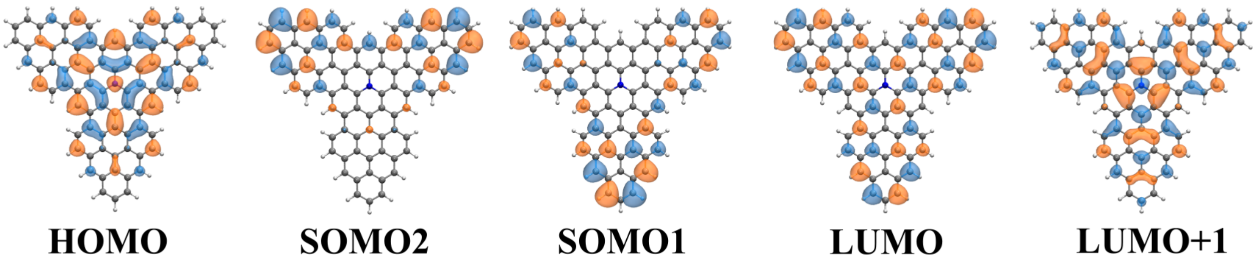

Figure S3: Frontier orbitals around the Fermi level used in CAS(11,11) obtained from the restricted open-shell Kohn-Sham (ROKS) DFT-PBE0 calculation for the **TTAT** molecule.

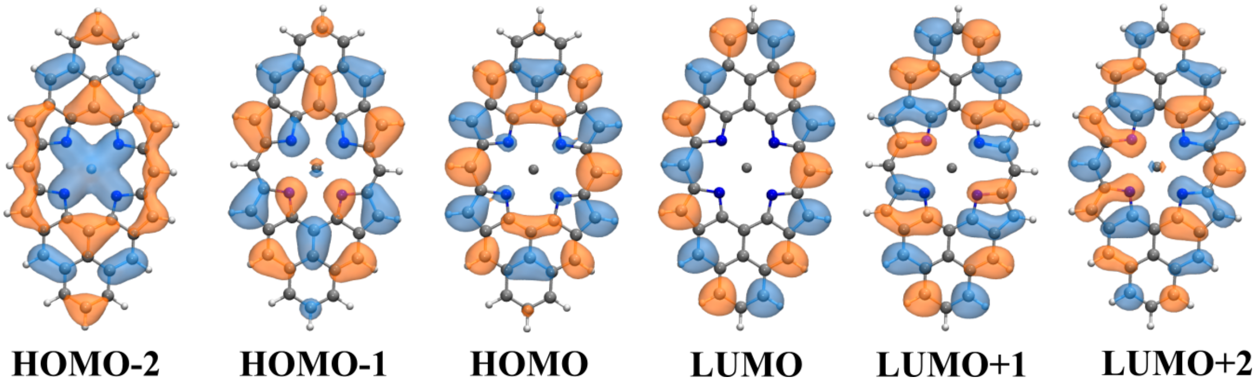

Figure S4: Frontier orbitals around the Fermi level used in CAS(12,12) obtained from the spin-unpolarized DFT-PBE0 calculation for the **APor**$_2$ molecule.

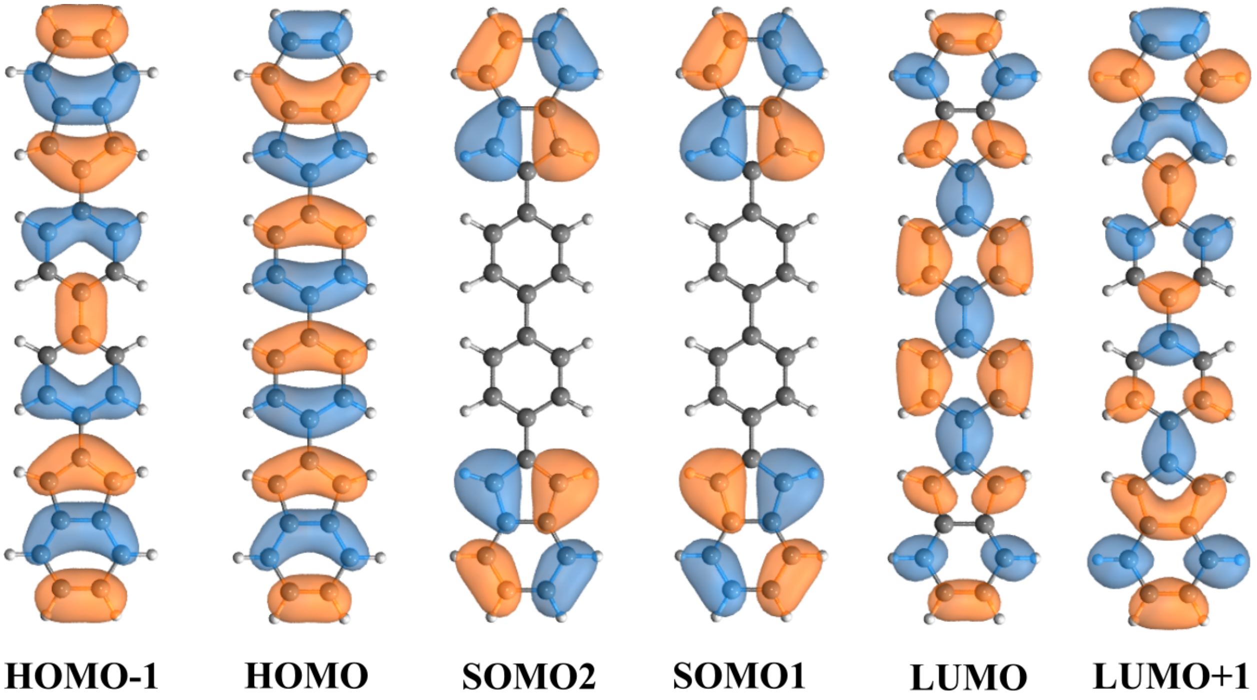

Figure S5: Frontier orbitals around the Fermi level used in CAS(12,12) obtained from the spin-unpolarized DFT-PBE0 calculation the **DNPAH** molecule.

6 Wavefunction Representation of the TTAT Molecule in Different S_z subspace

The wavefunction of the **TTAT** molecule in its neutral ground state can be represented in the $S_z = 1.5$ spin projection where it can be represented in a single Slater determinant. For charged states, the wavefunction can be described using an $S_z = 1.0$ representation. Importantly, the Dyson orbitals remain invariant with respect to the choice of S_z , indicating that the fundamental electronic transitions are independent of the spin projection selected for the description.

However, it is crucial to recognize that within the charge-state multiplets, there exist additional electronic states with $S_z = 0$ (corresponding to open-shell singlet configurations). These states are not included when constraining the Hamiltonian to a higher spin representation. Consequently, transitions involving single-electron processes that would typically manifest in Dyson orbitals become inaccessible due to the restriction imposed on the Hamiltonian. Specifically, any state with a total spin projection lower than the selected S_z of the Hamiltonian does not appear in the computed energy spectrum, as the Hamiltonian inherently excludes lower-spin configurations under these constraints.

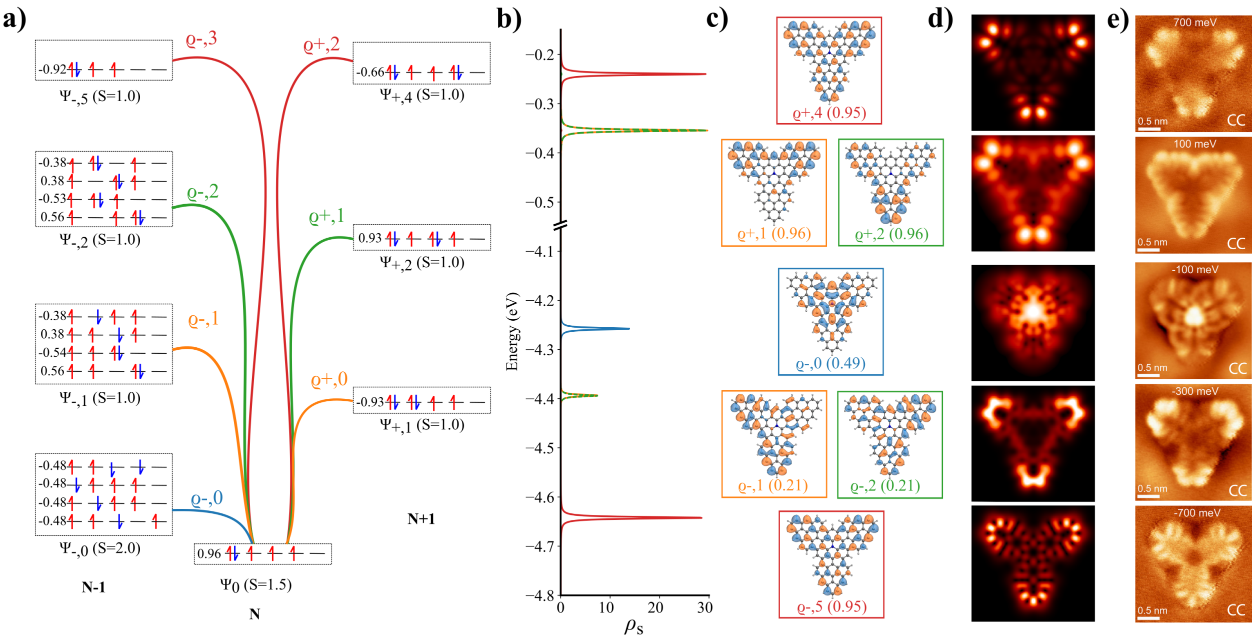

Figure S6: a) Multi-reference wavefunctions of neutral ground state (in $S_z = 1.5$ subspace) and charge state multiplets (in $S_z = 1.0$ subspace); b) calculated multi-reference spectral function; c) multi-reference Dyson orbitals obtained from CAS(11,11) calculations with corresponding strengths; d) simulated dI/dV of corresponding Dyson orbitals using PP-STM code[S5] with CO-like tip; e) experimental dI/dV maps adapted with permission from ref. Copyright 2025 American Chemical Society for **TTAT** molecule.

7 DMRG Dyson Orbitals of DNPAH diradical

We have also performed all-π CAS calculations to assess the quality of the lowest CAS(12,12) Dyson orbitals. Specifically, we carried out DMRG-SCF(30,30)/cc-pVDZ orbital optimization with a fixed DMRG bond dimension of $M = 1000$. Subsequently, we performed high-accuracy DMRG calculations for both the neutral and charged species, employing dynamical block state selection (DBSS)[S8] with a predefined truncation error of 10^{-6}. The resulting Dyson orbitals are presented in Figure S7. Orbital optimization was conducted using ORCA,[S4] while the DMRG calculations were performed with the MOLMPS program.[S9]

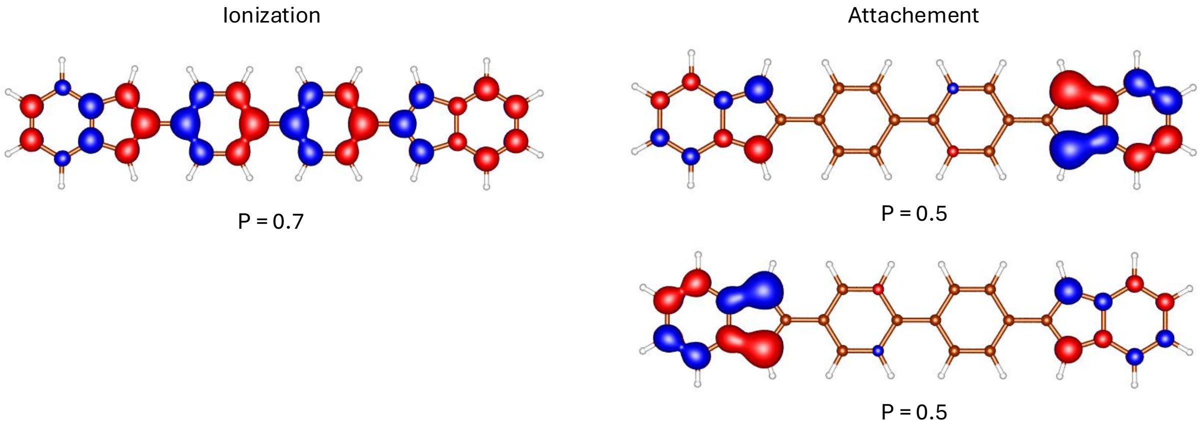

Figure S7: All-π Dyson orbitals (along with their probability factors) corresponding to the lowest-energy ionization and electron attachment processes were computed using the DMRG method. Note that the electron attachment Dyson orbitals are degenerate and therefore combine in the final STM signal.

References

(S1) Blum, V.; Gehrke, R.; Hanke, F.; Havu, P.; Havu, V.; Ren, X.; Reuter, K.; Scheffler, M. Ab initio Mol. Simul.s with numeric atom-centered orbitals. *Comput. Phys. Commun.* **2009**, *180*, 2175–2196.

(S2) Adamo, C.; Barone, V. Toward reliable density functional methods without adjustable parameters: The PBE0 model. *J. Chem. Phys* **1999**, *110*, 6158–6170.

(S3) Tkatchenko, A.; Scheffler, M. Accurate Molecular Van Der Waals Interactions from Ground-State Electron Density and Free-Atom Reference Data. *Phys. Rev. Lett.* **2009**, *102*, 073005.

(S4) Neese, F. The ORCA program system. *WIREs Comput Mol Sci* **2012**, *2*, 73–78.

(S5) Krejčí, O.; Hapala, P.; Ondráček, M.; Jelínek, P. Principles and simulations of high-resolution STM imaging with a flexible tip apex. *Phys. Rev. B* **2017**, *95*.

(S6) Truhlar, D. G.; Hiberty, P. C.; Shaik, S.; Gordon, M. S.; Danovich, D. Orbitals and the interpretation of photoelectron spectroscopy and (e, 2e) ionization experiments. *Angew. Chem.* **2019**, *131*, 12460–12466.

(S7) Andrews, J. S.; Jayatilaka, D.; Bone, R. G.; Handy, N. C.; Amos, R. D. Spin contamination in single-determinant wavefunctions. *Chem. Phys. Lett.* **1991**, *183*, 423–431.

(S8) Legeza, Ö.; Röder, J.; Hess, B. *Phys. Rev. B* **2003**, *67*, 125114.

(S9) Brabec, J.; Brandejs, J.; Kowalski, K.; Xantheas, S.; Legeza, Ö.; Veis, L. Massively parallel quantum chemical density matrix renormalization group method. *J. Comput. Chem.* **2021**, *42*, 534–544.
